# Device therapies for heart failure with reduced ejection fraction: a new era

**DOI:** 10.3389/fcvm.2024.1388232

**Published:** 2024-10-18

**Authors:** Rohit Mody, Abha Bajaj Nee Sheth, Debabrata Dash, Bhavya Mody, Ankit Agrawal, Inderjeet Singh Monga, Lakshay Rastogi, Amit Munjal

**Affiliations:** ^1^ Department of Cardiology, Mody Harvard Cardiac Institute & Research Centre, Krishna Super Specialty Hospital, Bathinda, India; ^2^ Department of Anatomy, Dr Harvansh Singh Judge Institute of Dental Sciences & Hospital, Panjab University, Chandigarh, India; ^3^ Department of Cardiology, Aster Hospital, Dubai, United Arab Emirates; ^4^ Department of Medicine, Kasturba Medical College, Manipal, India; ^5^ Department of Cardiology, Cleveland Clinic, Cleveland, OH, United States; ^6^ Department of Cardiology, Command Hospital Chandimandir, Panchkula, India; ^7^ Department of Cardiology, Dr Asha Memorial Multispecialty Hospital, Fatehabad, India

**Keywords:** congestive heart failure, implantable catheters, device, minimally invasive surgery, cardiac resynchronization

## Abstract

Even with significant advancements in the treatment modalities for patients with heart failure (HF), the rates of morbidity and mortality associated with HF are still high. Various therapeutic interventions, including cardiac resynchronization therapy, Implantable Cardiovascular-Defibrillators, and left ventricular assist devices, are used for HF management. Currently, more research and developments are required to identify different treatment modalities to reduce hospitalization rates and improve the quality of life of patients with HF. In relation to this, various non-valvular catheter-based therapies have been recently developed for managing chronic HF. These devices target the pathophysiological processes involved in HF development including neurohumoral activation, congestion, and left ventricular remodeling. The present review article aimed to discuss the major transcatheter devices used in managing chronic HF. The rationale and current clinical developmental stages of these interventions will also be addressed in this review.

## Graphical Abstract

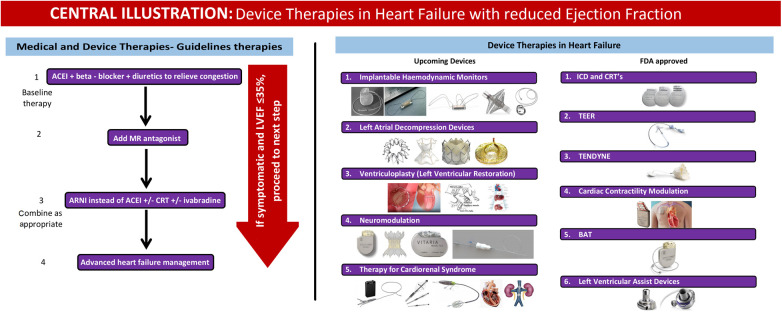

New catheter-based therapies beyond guideline directed therapy in heart failure.

## 1 Introduction

Heart Failure (HF) is a growing clinical syndrome characterized by neurohumoral system activation, leading to endothelial stress and chronic cardiac chamber remodeling, which heightens the risk for end-stage HF (1). HF is classified based on left ventricular ejection fraction (LVEF) into three major categories: heart failure with reduced ejection fraction [HFrEF; ejection fraction (EF) <40%], heart failure with preserved ejection fraction (HFpEF; EF >50%), and heart failure with mildly reduced ejection fraction (HFmrEF; EF 41%–49%). In addition, a new category, HF with improved EF, was introduced to account for patients with an increase in EF of >10% from baseline and an EF of >40% (2).

Approximately 6.7 million Americans aged over 20 years have HF, with projections indicating this will rise to 8.5 million by 2030 (3, 4). A 24% increase in the lifetime risk of HF has occurred, with one in four people expected to develop HF. Globally, around 56.2 million people have HF, and between 2010 and 2019 (5), the incidence rate increased by 29.4% (6). HFpEF is becoming more common, now representing over half of all HF cases. Despite a temporary decline in HF hospitalizations from 2010 to 2014, rates surged between 2014 and 2017 (7). Healthcare costs associated with HF are projected to exceed $60 billion by 2030 (8).

The neuroendocrine system activation in HF patients can lead to tachycardia and increased systemic vascular resistance. Sustained neurohumoral system activation (9), including the arginine vasopressin, sympathetic nervous, and renin–angiotensin–aldosterone systems (RAAS), contributes significantly to HF progression, exacerbating symptoms and driving arrhythmogenicity and cardiac remodeling. These systems are key targets for HF therapies (10). Treatment strategies vary, with some addressing the primary cause and others focusing on pharmacological and non-pharmacological interventions to curb HF progression and improve patients’ quality of life (QOL). Recent therapeutic advancements include angiotensin receptor–neprilysin inhibitors (11), RAAS antagonists, beta-blockers, cardiac resynchronization therapy (CRT), and sodium–glucose-cotransporter-2 inhibitors (12). Despite these advancements, HF morbidity and mortality remain high, with projections suggesting over 8 million people could develop HF by 2030, a 50% increase from 2012 (1).

New transcatheter diagnostic and therapeutic techniques, such as implantable hemodynamic monitors and left atrial decompression devices, target key pathways in HF development (13). As HFpEF prevalence rises, efforts continue to better categorize its phenotypes and refine diagnostic criteria. The review highlights the merits and limitations of device-based therapies in managing HF, HFpEF, and HFrEF.

The related devices can be divided into FDA-approved and upcoming devices.

**FDA-approved devices**

1. Implantable cardiovascular-defibrillators (ICDs) and CRT
2. Transcatheter edge-to-edge repair (TEER)
3. Tendyne System (Roseville, MN, USA)
4. Cardiac Contractility Modulation (CCM) (Impulse, Orangeburg, NY, USA)
5. Baroreflex activation therapy (BAT) (CVRs, Minneapolis, MN, USA)
6. Left ventricular assist devices (LVADs)

**Upcoming devices**

1. Implantable hemodynamic monitors
2. Left atrial decompression devices
3. LV restoration devices
4. Neuromodulation for the management of heart failure
5. Device-based therapy for cardiorenal syndrome

**Summary**

**Figure 1:**
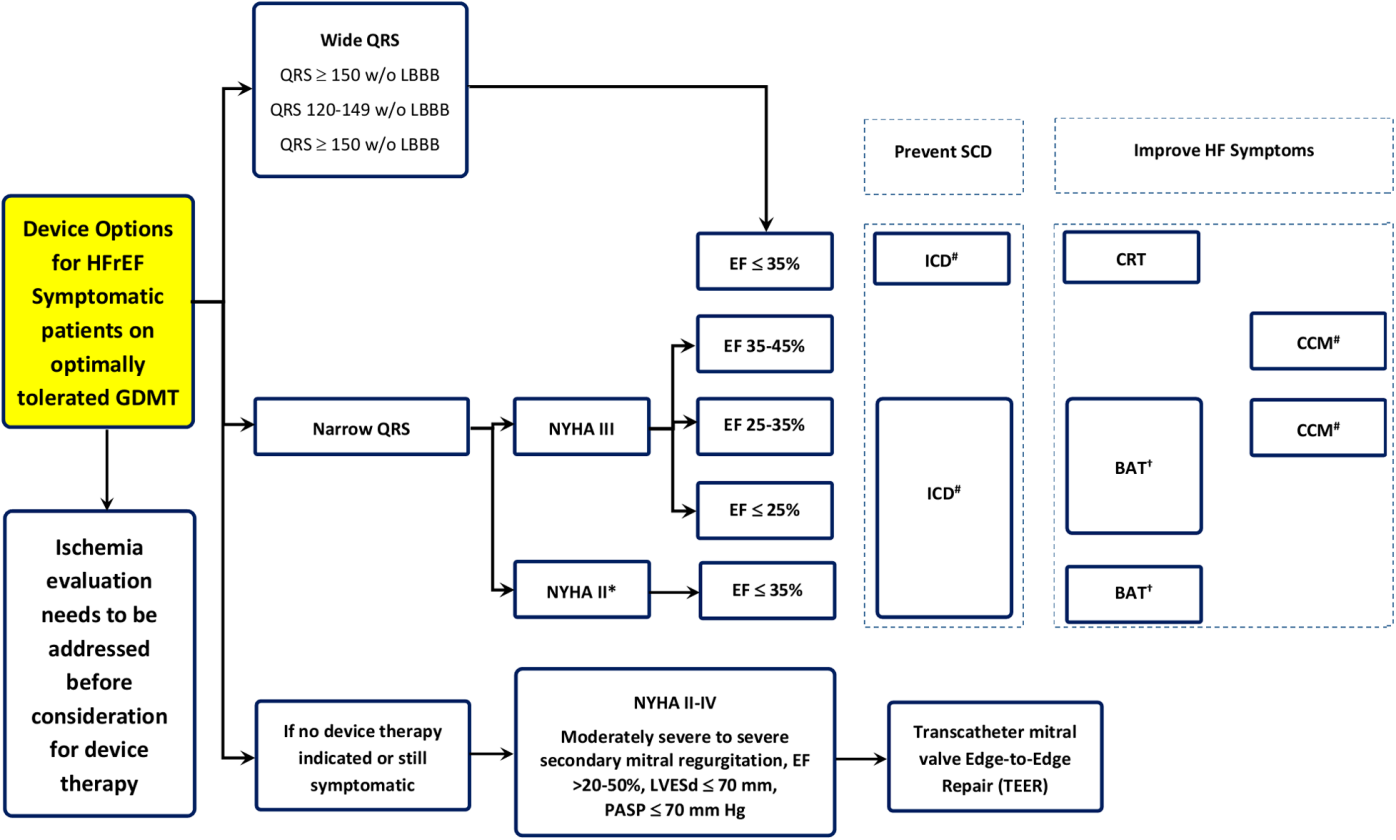
Therapeutic device options for the management of patients with HFrEF. ^†^BAT, baroreflex activation; ICD, implantable cardioverter–defibrillator; CCM, cardiac contractility modulation; SCD, sudden cardiac death; CRT, cardiac resynchronization therapy.

• Device therapy options for patients with HFrEF who are symptomatic despite guidelines-directed medical therapy (GDMT).
• The decision-making process starts with assessing ischemia before considering device therapy; if no device therapy is indicated or symptoms persist, different pathways are suggested based on QRS width.
• Patients with narrow QRS and New York Heart Association (NYHA) Class III are stratified by EF, where options like ICD, Barostim, and CCM are considered based on specific EF ranges (≤35%, 25%–35%, 35%–45%).
• Patients with a wide QRS are assessed for ICD or CRT therapy, depending on the QRS width and the presence of left bundle branch block (LBBB).
• The algorithm also includes options for patients with severe secondary mitral regurgitation (SMR), suggesting TEER for those with moderate to severe conditions and specific echocardiographic parameters. Therapeutic device options for patients with HFrEF are presented in Figure 1.

### 1.1 Food and Drug Administration–approved devices for HFrEF

**Figure 2:**
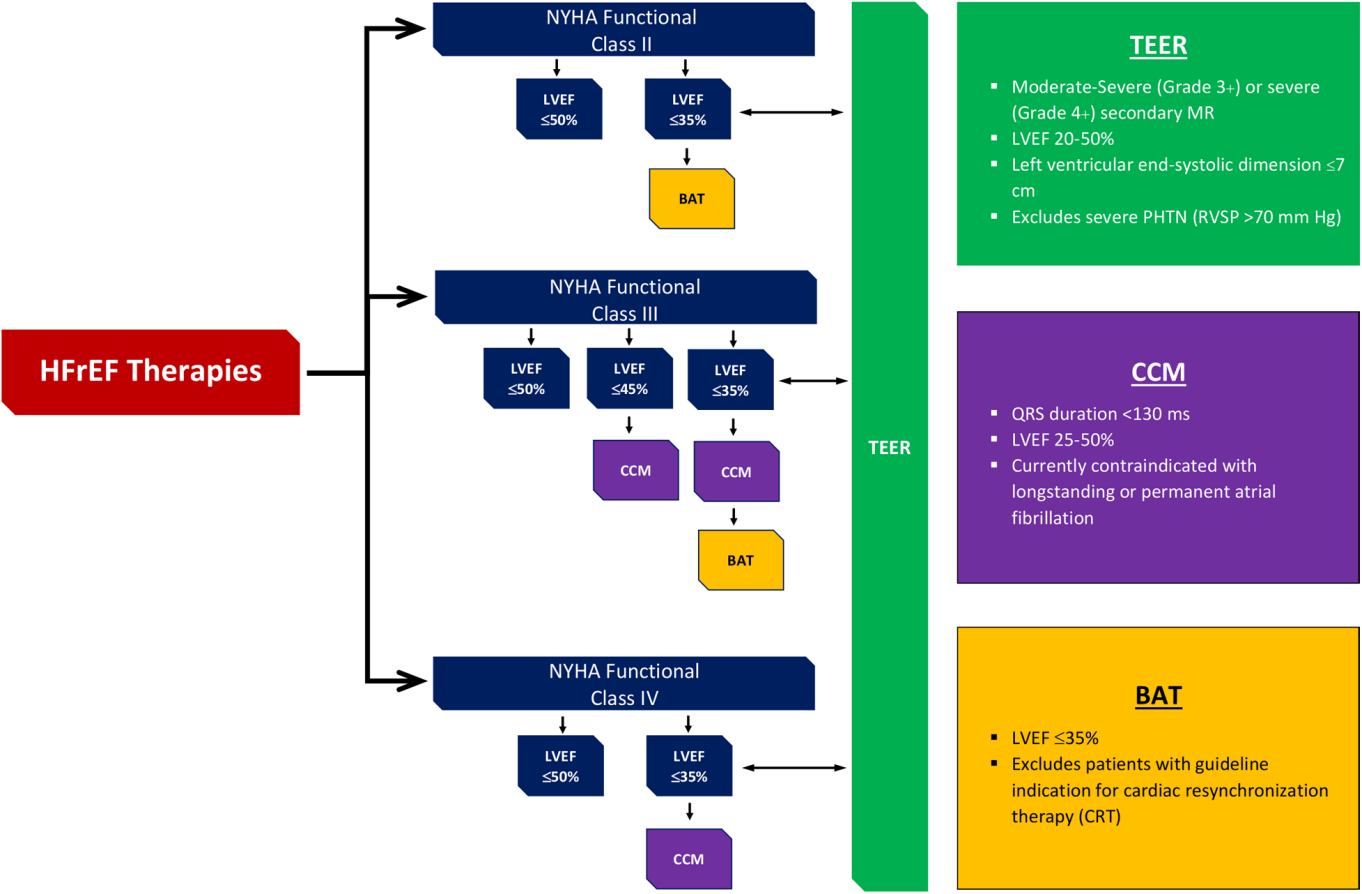
Devices approved by FDA to be used in HF along with their level of evidence according to NYHA class and LVEF. ^†^FDA, Food and Drug Administration; HF, heart failure; NYHA, New York Heart Association; LVEF, left ventricular ejection fraction; HFrEF, heart failure reduced ejection fraction; TEER, transcatheter edge-to-edge mitral valve repair; BAT, baroreflex activation therapy; CCM, cardiac contractility modulation.

Devices that have been approved by the Food and Drug Administration (FDA) to be used for prevention of HF according to NYHA class and LVEF are shown in Figure 2.

#### 1.1.1 Transcatheter edge-to-edge repair

TEER is a minimally invasive procedure used to treat mitral regurgitation (MR) by clipping the mitral valve leaflets together to reduce leakage. This technique is particularly beneficial for patients with HF who are not candidates for open-heart surgery.

##### 1.1.1.1 Clinical indications for mitral TEER

1. **Conventional indications**
  • **Secondary mitral regurgitation:** TEER is traditionally used to treat patients with functional MR due to dilated cardiomyopathy or HF.
  • **Degenerative mitral regurgitation:** It is also indicated for primary MR resulting from degenerative valve disease like mitral valve prolapse.
2. **Evolving indications**
  • **Advanced heart failure:** TEER is being explored as a treatment for patients with advanced HF who are not candidates for surgery.
  • **Failed surgical repair:** TEER is an option for patients who have had unsuccessful mitral valve surgery.
  • **Complex anatomy:** Cases involving complex mitral valve anatomy may also be treated with TEER.
  • **Post-myocardial infarction:** Patients who develop MR after a myocardial infarction (MI) are potential candidates for TEER.
  • **Congenital heart disease:** The use of TEER has been expanded to patients with congenital heart defects affecting the mitral valve.
  • **Hypertrophic obstructive cardiomyopathy (HOCM):** TEER is also being considered for managing MR in patients with HOCM (14).

TEER is considered a safe alternative for patients with severe MR who have surgical contraindications or high-operative risk (15). While the mitra clip device for functional regurgitation (MITRA-FR) trial showed no outcome improvement with TEER, the cardiovascular outcomes assessment of the mitra clip percutaneous therapy for heart failure patients with functional mitral regurgitation (COAPT) trial demonstrated a mortality benefit, leading to guideline recommendations for TEER in selected patients meeting COAPT criteria (16). TEER is particularly attractive for high-risk populations, including those with urgent mitral needs, complex mitral anatomies, or advanced HF although the supporting evidence varies across different patient groups, with stronger data for complex anatomy cases compared to congenital heart disease (17).

##### 1.1.1.2 TEER in patients with severe LV dysfunction or advanced HF

Patients with severely reduced left ventricular (LV) function and severe MR face poor outcomes, often similar to those in the MITRA-FR trial, where medical therapy was limited due to comorbidities (18). TEER's role in these patients is yet undetermined, with most data coming from registries like the transcatheter mitral valve interventions (TRAMI) registry, which showed significant clinical benefits during a 1-year follow-up in patients with LV ejection fraction <30% undergoing TEER with MitraClip. A recent study also demonstrated marked improvement in NYHA functional class and QOL in NYHA Class IV patients treated with MitraClip, although 1-year mortality and HF hospitalization rates remained high. The MitraBridge registry highlighted MitraClip as a safe bridge to heart transplantation in patients with severe SMR and advanced HF, although clinicians must carefully weigh the risks and benefits of each therapy option, including mechanical support and heart transplantation (19).

#### 1.1.2 Tendyne system

The Tendyne transcatheter mitral valve repair (TMVR) system by Abbott (Atlanta, GA, USA) is a self-expanding, intra-annular nitinol bioprosthesis with a trileaflet porcine pericardial valve, designed to prevent paravalvular MR with its supra-annular cuff (20). It is delivered transapically via a 36F sheath and anchored to the left ventricular apex, with the option to adjust the tether post implantation to reduce paravalvular leaks. The system has been studied extensively, with a cohort of 100 patients showing a 96% technical success rate and 72.4% 1-year survival (21). A pivotal randomized trial is underway Study to understand mortality and morbidity in COPD (SUMMIT) trial compares Tendyne TMVR with TEER using MitraClip, focusing on 1-year survival free of HF hospitalization and other cardiovascular events (22). The device has been approved for CE mark in Europe since 2020.

#### 1.1.3 Optimizer, a CCM system

CCM is a device-based therapy for chronic HF gaining in popularity, and now integral to the European Society of Cardiology Guidelines (23). The optimizer smart system, a CCM device, alters the myocardial wall properties by delivering high voltage, prolonged electrical signals to the right ventricular (RV) septum, enhancing contractility, and reversing ventricular remodeling. Initially designed for patients with HFrEF unsuitable for CRT, the most recently conducted FIX-HF-5C confirms its safety and efficacy. This trial highlighted improved peak oxygen consumption, 6-Minute Walking Distance (MWD) score, and QOL, and decreased HF-related hospitalizations and mortality when using the device (24).

**Cardiac contractility modulation signals**

• CCM signals are non-excitatory, biphasic, high-voltage pulses applied during the absolute refractory period.
• Therapy is delivered in seven 1-h sessions equally spaced over 24 h, with the device recharged for 1 h on a weekly basis.
• The CCM device's rechargeable battery is warranted for 20 years.

**Cardiac contractility modulation therapy**

• CCM therapy is delivered by an implanted pulse generator with a rechargeable battery and two RV septal leads.
• CCM signals modulate the biology of the failing myocardium, improving contractility without increasing myocardial oxygen consumption.
• The therapy results in improved peak volume of oxygen (VO_2_) and QOL, and reduced HF hospitalizations.

**Cardiac contractility modulation therapy effects**

• CCM rapidly improves contractility within minutes to hours by enhancing calcium cycling and protein phosphorylation without increasing oxygen consumption.
• The intermediate effects over hours to weeks include the normalization of the gene profile, shifting from a HF gene program to a more normal state.
• Long-term effects over weeks to months involve beneficial global ventricular changes and reverse remodeling of the heart.

**Cardiac contractility modulation randomized trials**

• The FIX-HF 4 trial showed a significant improvement in peak VO_2_ (+0.99) after 3 months with CCM therapy.
• The FIX-HF 5 trial demonstrated an increase in peak VO_2_ (+0.65) over 6 months, although the primary endpoint was the anaerobic threshold.
• The FIX-HF-5C trial reported improved exercise tolerance with a +0.84 increase in peak VO_2_ for EF 25%–45% and +1.67 for the subgroup with EF 35%–45% after 6 months of CCM therapy.

CCM therapy, demonstrated through the FIX-HF trials, significantly improves peak VO_2_, exercise tolerance, and QOL in patients with HF, particularly those with EF 25%–45%. The FIX-HF-5C study confirmed these benefits, showing significant improvements in the 6-MWD Minnesota Living with Heart Failure Questionnaire (MLWHFQ) score and NYHA class, with a substantial reduction in cardiovascular deaths and HF hospitalizations. The trials concluded that CCM is a safe and effective treatment for severe HF, leading to better functional outcomes and fewer hospitalizations.

**CCM device indication**

• CCM is indicated to improve functional status (NYHA class), 6-MWD, and QOL
• The FDA approved the CCM therapy on 21 March 2019, for patients with NYHA Class III HF who remain symptomatic despite GDMT.
• CCM is approved for patients not indicated for CRT with an LVEF ranging from 25% to 45%.

**Future directions**

**Figure 3:**
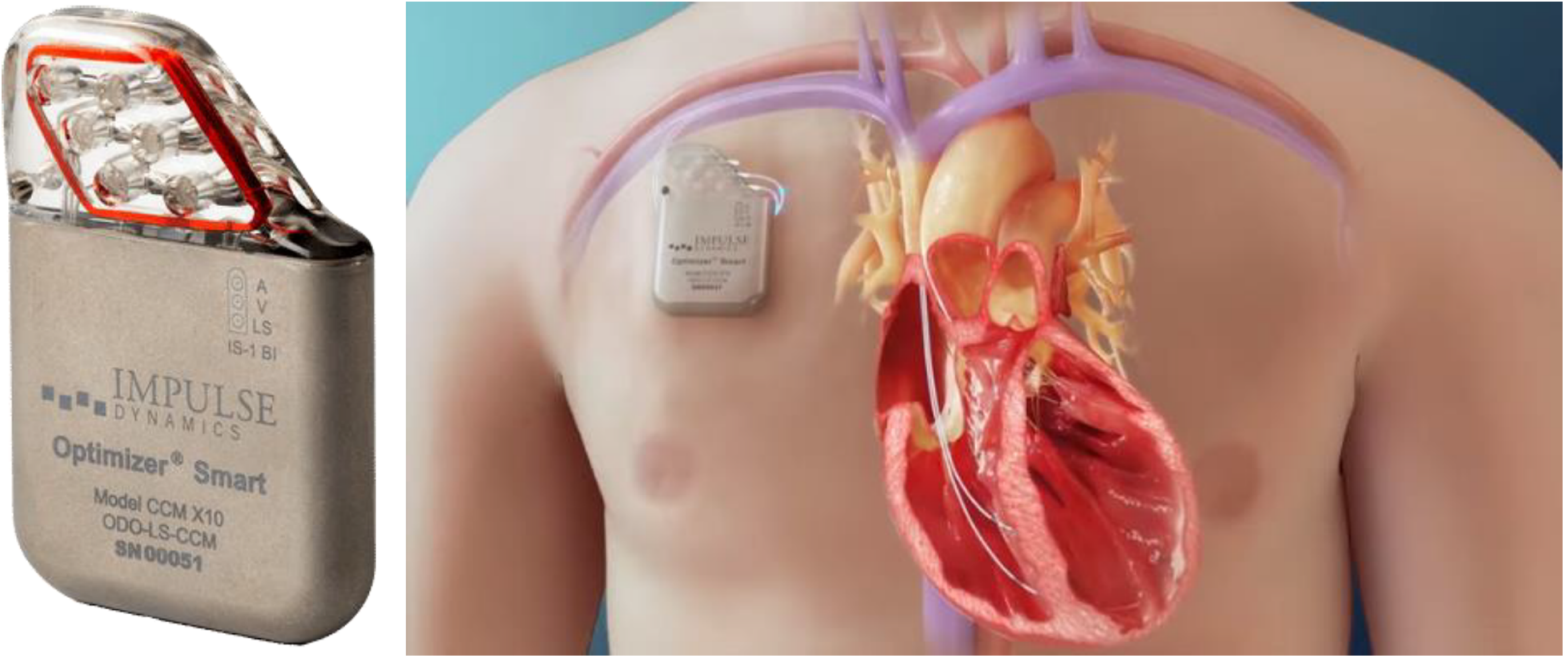
Optimizer cardiac contractility modulation system used for heart failure management.

• The Post Approval Study includes 620 subjects with a 3-year follow-up focusing on MLWHFQ, mortality, and safety (25).
• The AIM HIGHER Trial, initiated in February 2022, involves around 1,500 subjects with LVEF 40%–60%, using a randomized, double-blinded design with 6- and 18-month endpoints (26).
• The INTEGRA-D Trial, starting in early 2023, involves 300 subjects and has the FDA Breakthrough Designation, with 6-month and 2-year endpoints (27). The optimizer system shows promise for chronic HF management. Optimizer CCM used for managing chronic HF is shown in Figure 3.

#### 1.1.4 Baroreflex activation therapy

BAT has emerged as an innovative therapeutic approach for HF management that includes baroreceptor electrical stimulation. This therapeutic strategy potentiates the autonomic nervous system activity, reducing the sympathetic activity and modulating the activity of the parasympathetic nervous system. Marked improvements in symptoms of HF, QOL, and functional capacity were seen in patients undergoing BAT per the findings of clinical trials, such as barostim hope of heart failure study and baroreflex activation therapy for heart failure. Further, marked decline in HF hospitalization rate and improvement in exercise tolerance were reported in the results of the BeAT-HF. BAT can be utilized as a promising adjunctive therapeutic approach for patients with HF, specifically for patients who remain asymptomatic, instead of optimal medical therapy (28).

**Autonomic nervous system in heart failure**

• HF leads to decreased baroreceptor signaling, resulting in reduced baroreflex sensitivity.
• This causes an imbalance in the autonomic nervous system, with increased sympathetic and decreased parasympathetic activity.
• The autonomic imbalance contributes to adverse effects such as increased heart rate, reduced diuresis, enhanced renin secretion, vasoconstriction, and elevated blood pressure.

**Electrically loading the baroreflex**

• Barostim therapy electrically stimulates the carotid baroreceptors to rebalance the autonomic nervous system.
• This stimulation inhibits sympathetic activity and enhances parasympathetic activity, leading to improved heart function.
• The therapy results in reduced heart rate and cardiac remodeling, increased vasodilation, and improved diuresis and blood pressure control.

**Summary**

• Barostim clinical trials evolved from safety assessments in Phase I to demonstrating safety and efficacy in Phase II and the pivotal BAT in Beat-HF trial, which involved 408 subjects.
• The BeAT-HF trial's Pre-Market Phase focused on exercise capacity, QOL, NYHA class improvement, and N-terminal pro B-type natriuretic peptide (NT-pro-BNP) reduction in 264 patients.
• Following FDA approval in 2019, the Post-Market Phase of BeAT-HF included 59 additional patients, emphasizing cardiovascular mortality, HF morbidity, and safety.
• BeAT-HF inclusion criteria required NYHA Class III heart failure patients with LVEF ≤35%, stable on optimal medical therapy, and at elevated risk for clinical events.
• Symptomatic improvements at 6 months in BeAT-HF showed a 60-min increase in exercise capacity and a 14-point improvement in QOL with Barostim.
• The BeAT-HF trial demonstrated a significant 25% reduction in NT-pro-BNP levels, surpassing the clinically meaningful 10% reduction benchmark.
• Barostim therapy achieved a 97% major adverse cardiovascular events (MACE)–free rate, indicating low neurological event risks within 6 months post implantation.
• Barostim significantly reduced serious cardiovascular events by 51% compared to the control, with lower event rates for arrhythmias, MI/angina, and hypotension/syncope.

**BAROSTIM system indication**

• The BAROSTIM NEO system, which provides BAT, was FDA approved on 19 August 2019.
• It is indicated for patients with NYHA Class II/III HF, LVEF ≤35%, and NT-pro-BNP <1,600 pg/ml who remain symptomatic despite GDMT.
• The system is not indicated for patients eligible for CRT according to American College of Cardiology (ACC)/American Heart Association (AHA)/European Society of Cardiology (ESC) guidelines.

**BeAT-HF results: post-market phase**

• The BeAT-HF post-market phase showed a 97% MACE-free rate and significant long-term improvements in exercise capacity, QOL, and NYHA class with Barostim therapy.
• The trial found no statistically significant difference in primary mortality endpoints but suggested a 34% relative reduction in all-cause mortality with Barostim (*p* = 0.054).
• These findings are based on a presentation and have not yet been peer-reviewed or published in a medical journal.

**BeAT-HF summary of key evidence**

• The BeAT-HF trial showed that Barostim therapy had a favorable impact on all-cause mortality with a hazard ratio of 0.66, and a positive hierarchical win ratio of 1.26 for cardiovascular outcomes and QOL (28).
• Barostim significantly improved long-term safety, with a 96.9% MACE-free rate, and enhanced symptoms, including QOL, exercise capacity, and NYHA class improvement.
• The primary endpoint showed a slight favoring of Barostim for cardiovascular mortality but a neutral impact on HF morbidity, with a rate ratio of 0.94 for mortality and 1.08 for morbidity.

#### 1.1.5 ICDs and CRT-Ds

For the clinical use in HF, both ICDs and CRT with defibrillators (CRT-Ds) have got the class of Recommendation I and level of Evidence A (as described in Figure 1 and summary).

##### 1.1.5.1 Protect-ICD

Sudden cardiac death (SCD) is a leading cause of mortality after acute MI, particularly within the first three months. Current guidelines recommend ICD implantation only after this period, relying solely on LVEF for risk stratification, which lacks sensitivity and specificity. Patients with LVEF 31%–40% and no HF symptoms are also not currently considered for ICDs, despite a significant SCD risk. An international trial is exploring the use of electrophysiology studies (EPS) for early post-MI risk stratification, potentially enabling earlier ICD implantation for those at highest SCD risk (29).

##### 1.1.5.2 PROFID ICD trial

The PROFID EHRA trial is currently ongoing (started on November 2023) and will complete in 2.5 years, providing ICD clinical use justification for primary prevention in HF. SCD remains a leading cause of mortality, often following MI. While ICDs have reduced SCD risk in patients with severely reduced EF, the majority of SCD cases occur in those with moderately reduced or preserved ejection fraction. The PROFID project aims to revolutionize SCD risk stratification by developing a personalized prediction model through data analysis and validating it in large clinical trials. This approach seeks to improve ICD decision-making and reduce unnecessary implantations, potentially transforming SCD prevention across the full range of ejection fractions (30).

##### 1.1.5.3 Extravascular ICD pivotal study

In the findings of the prospective global study, we reported that extravascular ICDs were safely implanted in patients and were associated with the potential to identify and terminate induced ventricular arrhythmia at the implantation time (31).

##### 1.1.5.4 Budapest CRT trial

With the objective of demonstrating the efficacy of the CRT-D upgrade, a multicenter, randomized, and controlled trial was conducted in which 360 patients with HFrEF with a pacemaker and cardioverter–defibrillator were randomly assigned to receive CRT-D at the follow-up of 12 months. The primary clinical outcomes were the composite of HF hospitalization, all-cause mortality, and <15% decline of left ventricular end-systolic volume. In the findings of the study, reduced combined risk of all-cause mortality and HF hospitalization were reported in patients upgraded to CRT-D as compared to ICD (32).

**Figure 4:**
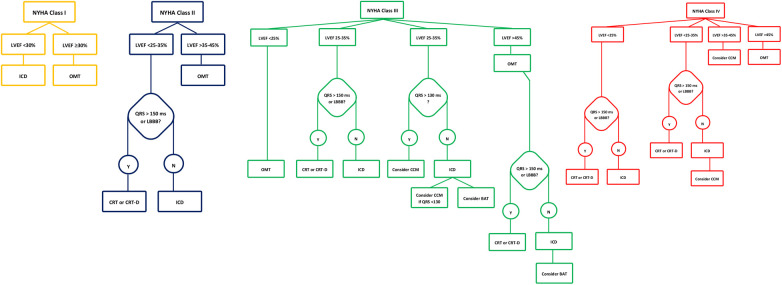
Device-based algorithm for the management of patients with heart failure and reduced ejection fraction. Devices approved by FDA to be used in HF along with their level of evidence according to NYHA class and LVEF. ^†^FDA, Food and Drug Administration; HF, heart failure; NYHA, New York Heart Association; LVEF, left ventricular ejection fraction; HFrEF, heart failure reduced ejection fraction; TEER, transcatheter edge-to-edge mitral valve repair; BAT, baroreflex activation therapy; CCM, cardiac contractility modulation.

The Budapest CRT Upgrade trial is an international randomized controlled trial comparing the outcomes of CRT-D to ICD upgrades in patients with HFrEF with previously implanted pacemakers or ICDs and RV pacing ≥20%. The study enrolled 360 patients, randomly assigning them to either CRT-D or ICD upgrades. The primary endpoint was the composite of all-cause mortality, HF hospitalization, or a ≥15% reduction in left ventricular end-systolic volume (LVESV) within 1 year. The trial demonstrated that CRT-D significantly reduced the incidence of the primary endpoint and secondary composite outcomes, including all-cause mortality and HF hospitalizations, compared to ICD alone. The hazard ratio for all-cause mortality or heart failure hospitalization was significantly lower in the CRT-D group, indicating a substantial benefit in upgrading to CRT-D in these patients (33). The device-based algorithm for the management of patients with HF and reduced EF is demonstrated in Figure 4.

#### 1.1.6 LVADs

##### 1.1.6.1 LVAD as destination therapy

Mechanical circulatory support offers a viable alternative for managing advanced HF in patients ineligible for transplantation, significantly enhancing functional capacity and survival rates. With improvements in implantable pumps, specialized non-transplant LVAD centers have emerged, leading to better outcomes, with survival rates over 70% at 2 years and 50% at 5 years. However, short- and mid-term success depends on careful preimplant patient selection, emphasizing the importance of evaluating frailty, renal function, and hemodynamics. A multidisciplinary, shared-decision approach is crucial, especially for patients with comorbidities or uncertain prognosis (34).

##### 1.1.6.2 Bridge to recovery and decision

Patients with dilated cardiomyopathy and refractory end-stage HF have shown significant cardiac and physical recovery with combined LVAD and medical therapies, sometimes reaching levels comparable to healthy individuals. Encouraging more aggressive strategies for cardiac remodeling during LVAD support could increase the potential for LVAD explanation and a return to pharmacological management. Early identification of myocardial recovery can be achieved through central hemodynamic measurements during cardiopulmonary exercise testing in patients with LVAD (35).

##### 1.1.6.3 Bridge to transplantation

LVADs, when used as a bridge to transplantation, significantly enhance survival rates and QOL in patients with advanced congestive HF who are unresponsive to medical therapy (36). LVADs have emerged as highly effective tools for managing end-stage HF, offering superior clinical outcomes compared to traditional treatments. These devices are designed to offload the LV and modulate cardiac output (CO), leading to substantial improvements in the outcomes of patients with HFrEF, including increased survival rates and reduced mortality (37). The primary objective of an LVAD is to continuously unload the LV and provide a substantial physiological CO. However, current evidence on the use of LVADs in patients with HFpEF, who typically have smaller LV sizes, remains limited. The HeartMate 3 device, now widely used in the United States, has addressed the issue of pump thrombosis seen with the HeartMate II. These devices drain blood from the LV apex, returning it to the arterial system, and often result in enhanced kidney function post implantation. However, the potential for acute kidney injury due to post-operative RV failure necessitates careful risk assessment. Involving a nephrologist in the LVAD care team may optimize clinical outcomes (38).

##### 1.1.6.4 Impella—the percutaneous LVAD

**Figure 5:**
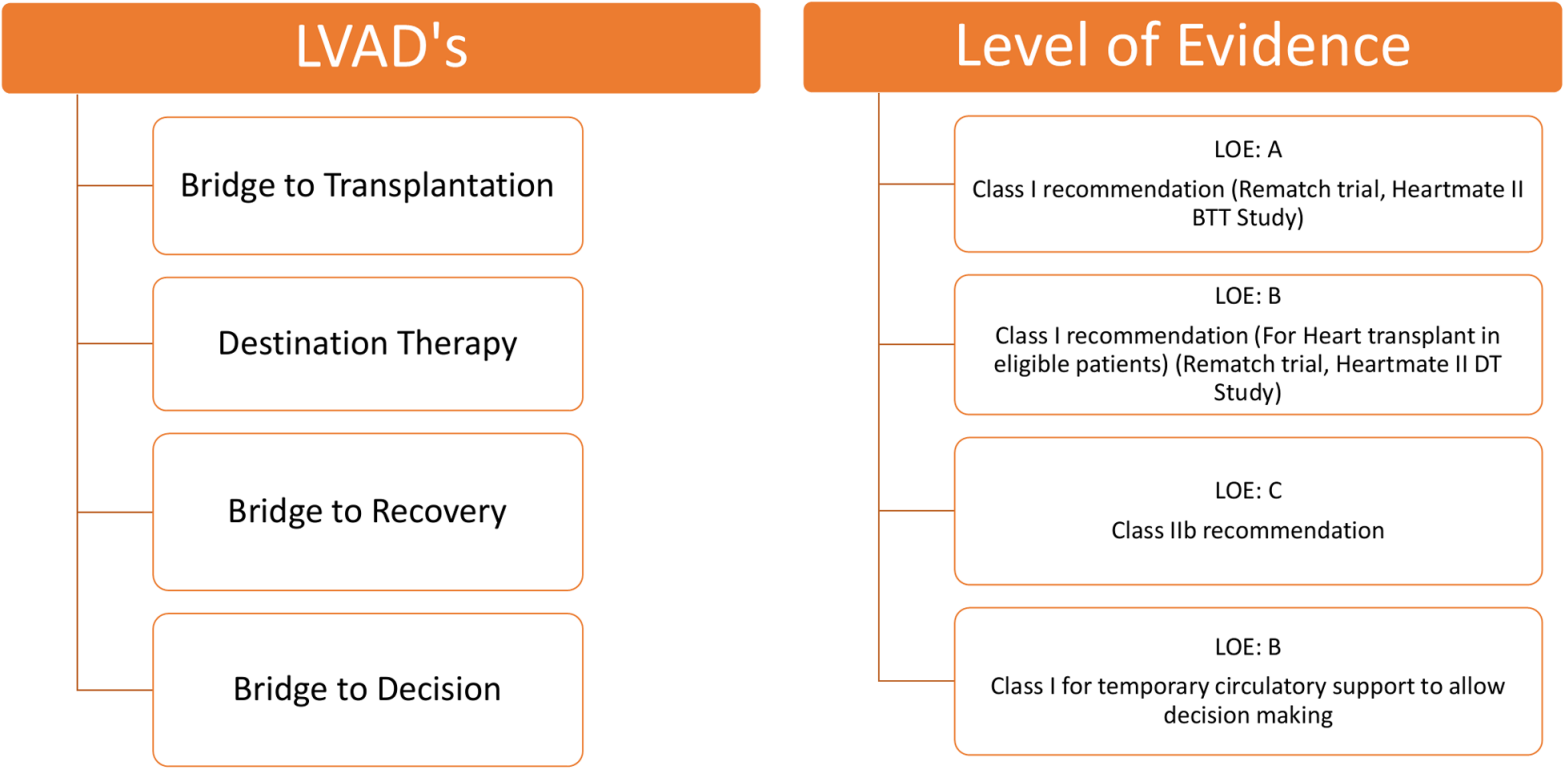
Level of evidence for clinical use of LVADs according to class of recommendation and LOE to clinical strategies, interventions, treatments, or diagnostic testing in patient care (updated May 2019). ^†^LVAD, left ventricular assist device; LOE, level of evidence.

Another percutaneous LVAD, the Impella, enhances systemic circulation and improves end-organ function in patients with cardiogenic shock (CS). The Impella is primarily inserted percutaneously, unloading the LV by transferring blood from the LV to the ascending aorta (39). Various randomized controlled trials have shown that, in patients with CS, the use of the Impella has not demonstrated a significant survival advantage compared to the intra-aortic balloon pump (40). However, when implanted at an optimal time and managed appropriately, the Impella provides significant circulatory support in a minimally invasive manner. The FDA has designated the Impella CP with SmartAssist heart pump as a safe and effective device for clinical use during high-risk percutaneous coronary intervention procedures and in patients with CS. It is a minimally invasive, temporary heart pump that utilizes real-time intelligence to improve patient survival rates and heart recovery. To evaluate the efficacy of the microaxial flow pump (Impella CP) on the mortality of patients with ST-elevation MI complicated by CS, an international, multicenter, randomized trial (the DanGer Shock trial) enrolled 360 patients. At the 180-day follow-up, the primary endpoint was death from any cause. The study results indicated that the mortality rate was lower in the pump group (45.8%) compared to the standard care group (58.5%). However, the incidence of adverse events, such as severe bleeding, limb ischemia, hemolysis, and the need for renal replacement therapy, was higher with the use of the Impella CP (41). The level of evidence supporting the clinical use of LVADs is shown in Figure 5.

##### 1.1.6.5 Autoregulated Carmat, Vélizy-Villacoublay, Paris-region

**Figure 6:**
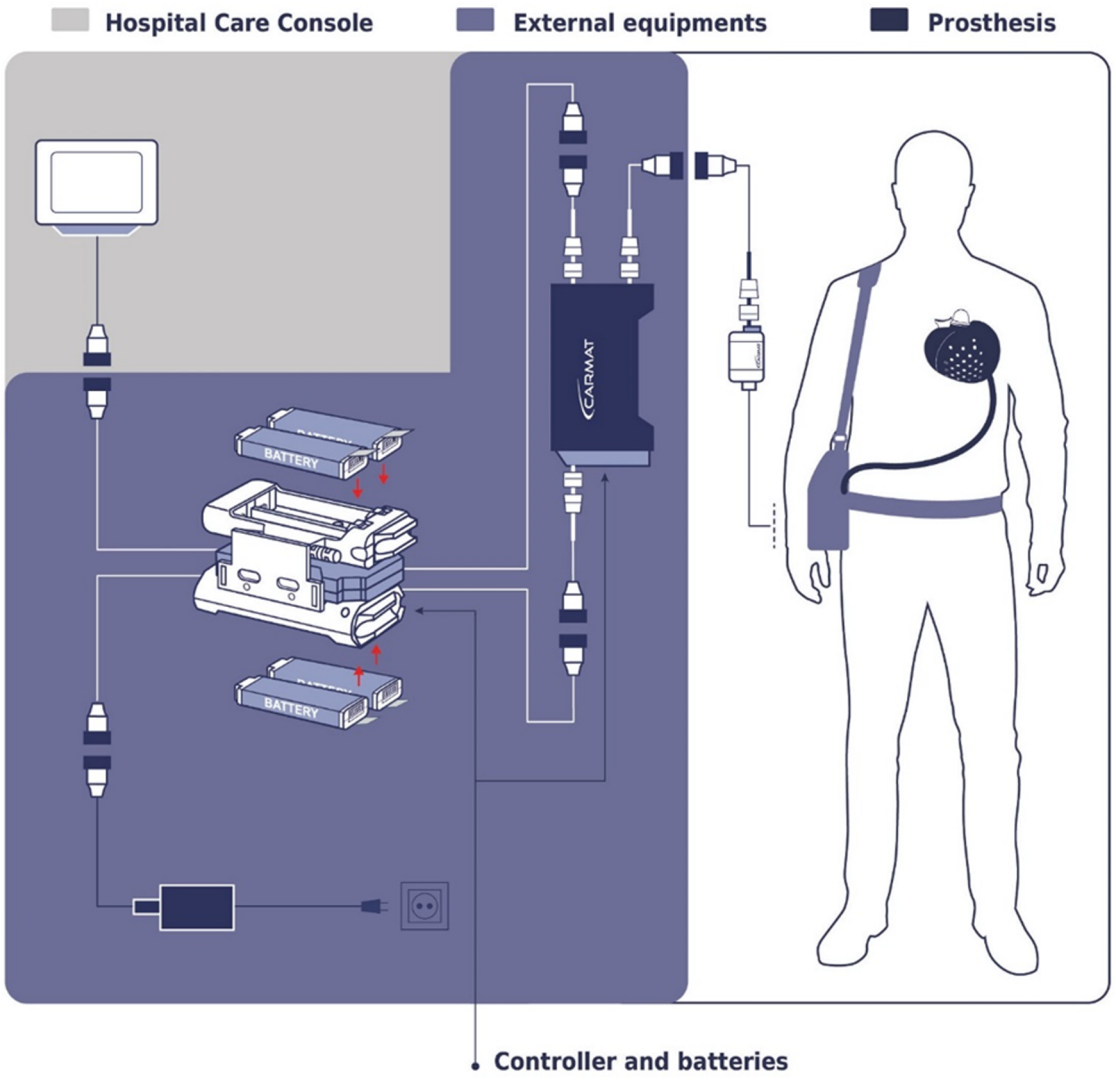
The workflow of CARMAT-total artificial heart.

In an article, Netuka et al. discusses the clinical experience with the CARMAT-Total Artificial Heart (C-TAH), highlighting its autoregulation system designed to emulate physiological heart functions in patients with end-stage biventricular failure. The device utilizes pressure sensors to adjust CO automatically based on venous return, providing nearly physiological heart replacement therapy. The study, involving 10 patients, demonstrated that the C-TAH's auto-mode effectively maintained stable hemodynamics with minimal need for manual adjustments, suggesting its potential for improving patient outcomes with reduced clinical intervention. The findings underscore the device's capability to support long-term recovery and enhance QOL for patients outside of the hospital setting (42). The workflow of CARMAT-Total Artificial Heart is shown in Figure 6.

### 1.2 Right ventricular assist devices

Acute right-sided HF (RHF) is a clinical syndrome associated with an increased incidence of morbidity and mortality. In patients with severe right ventricular dysfunction, temporary right ventricular assist devices (T-RVAD) have emerged as a potential therapeutic option, a permanent solution, and a bridge-to-recovery option. Durable RVAD can be used to reverse the pathophysiological mechanisms, adaptive remodeling, and impaired myocardial contractility involved in RHF progression (43). The most commonly used RVAD devices are Impella RP (Abiomed, Danvers, MA, USA), ProtekDuo (TandemLife, Pittsburg, PA, USA), TandemHeart (TandemLife, Pittsburg, PA, USA), and CentriMag (Levitronix LLC, Waltham, MA, USA). ProtekDuo is a 29-F or 31-F dual-lumen cannula used for creating a T-RVAD with or without an oxygenator by connecting it to other various extracorporeal devices (44). Under fluoroscopic and ultrasound guidance, this device is inserted percutaneously through the right internal jugular vein. Further, the Impella RP is a catheter-based microaxial pump that is positioned across the tricuspid and pulmonary valves and inserted in the femoral vein (45). Another extracorporeal centrifugal-flow pump used is the T-RVAD, which is also used in treating RHF. Most commonly, both femoral veins were used for the successful implantation of T-RVAD. The clinical use of T-RVAD with ProtekDuo has been demonstrated in seven studies (46).

Impella RP is surgically implanted through a sternotomy. One study utilized Impella RP in a cohort of 15 patients (47). Recently, the Impella RP (Abiomed, Danvers, MA, USA), a single vascular access, percutaneous, minimally invasive device approved by the FDA, has been introduced for the treatment of RVF. The prospective, open-label, and non-randomized multicenter RECOVER RIGHT study has demonstrated the safety and efficacy of the Impella RP. This study has reported considerable improvement in patient hemodynamics immediately after the initiation of Impella RP with a marked increase in the cardiac index and reduction in central venous pressure (CVP) (48). Further, the Impella RP flex, which reduces the risk of RV distortion and occupies less space in the superior vena cava, emerged as a new and safe therapeutic option for RV dysfunction. Recently, it was approved by the FDA as a mechanical circulatory support device for the RV (49).

### 1.3 Upcoming devices for HF

**Figure 7:**
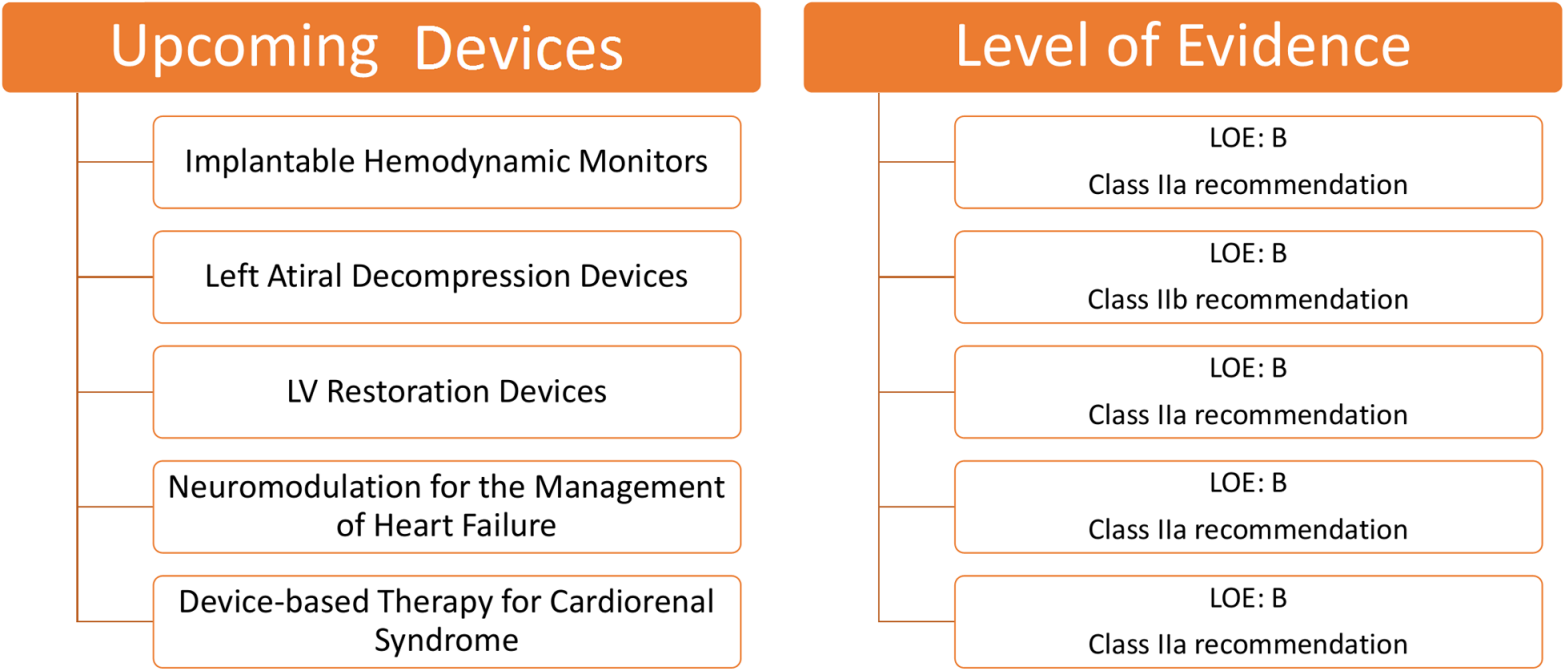
Upcoming devices for HF and their level of evidence for clinical use according to class of recommendation and LOE to clinical strategies, interventions, treatments, or diagnostic testing in patient care (updated May 2019).^†^HF, heart failure; LOE, level of evidence.

Upcoming devices for HF and their level of evidence for clinical use are shown in Figure 7.

#### 1.3.1 Implantable hemodynamic monitors

Patients with HF commonly experience deteriorating health, leading to unplanned hospitalizations. This decline often results from increased pressure in the left atrium (LA) and problems, such as peripheral and pulmonary congestion (50). Intriguingly, an increase in RV pressure and pulmonary artery pressure (PAP) can be observed before overt HF symptoms emerge during acute decompensation.

A considerable advancement in implantable devices that facilitate remote monitoring has been reported. These devices measure left atrial pressure (LAP) or PAP, aiding in the early identification of potential HF complications. Their monitoring capabilities have notably decreased the hospitalization rates for patients with HF.

The Chronicle by Medtronic Inc. (Minneapolis, MN, USA) is a device resembling an implantable pacemaker. Positioned in the pectoral muscles and linked via a transvenous lead, it constantly records the heart rate and RV pressure, transmitting this data for remote access. However, the cardiovascular outcomes for people using anticogulation strategies trial highlighted a challenge; despite its benefits, the Chronicle had an 8.5% complication rate and did not considerably diminish the HF-related adverse outcomes, underlining the need for device improvement and further research (51).

Various other devices are gaining attraction in the field of PAP monitoring. The CardioMEMS™ HF System by Abbott (Atlanta, GA, USA) is particularly notable. This device facilitates real-time monitoring of PAP in patients with HF. The CHAMPION trial reported a 37% reduction in the hospitalization risks using CardioMEMS, with only a 1.4% device-related complication rate (52). Further studies reinforced its benefits, indicating up to a 57% decrease in HF-related hospitalizations post implementation (53). The MONITOR-HF study subsequently affirmed a 44% reduction (54).

Other considerable devices include the Cordella™ PA Pressure Sensor System, V-LAP™ (Vectorius, Los Angeles, CA, USA), and HeartPOD (Carmat, Vélizy-Villacoublay, France). The Cordella™ PA Pressure Sensor System comprises a PA sensor, a Cordella delivery system, and a handheld reader for the continuous assessment of PAP (1). Marked improvements in the NYHA functional class and QOL were seen in an early feasibility study conducted on 15 patients receiving medical therapy guided by the Cordella system. Device-related complications were not seen in this study (55). Further, the accuracy and safety of invasive PAP monitoring by Cordella™ PA Pressure Sensor System have been demonstrated in a recently published CE-Mark SIRONA-2 trial (56). The Cordella system not only observes the hemodynamic shifts but also assesses the vital signs. V-LAP™ uses artificial intelligence to enhance its efficiency. Despite its initial promise, the HeartPOD system encountered challenges in the left atrial pressure monitoring to optimize heart failure therapy trial due to implant-related complications (57).

**Figure 8:**
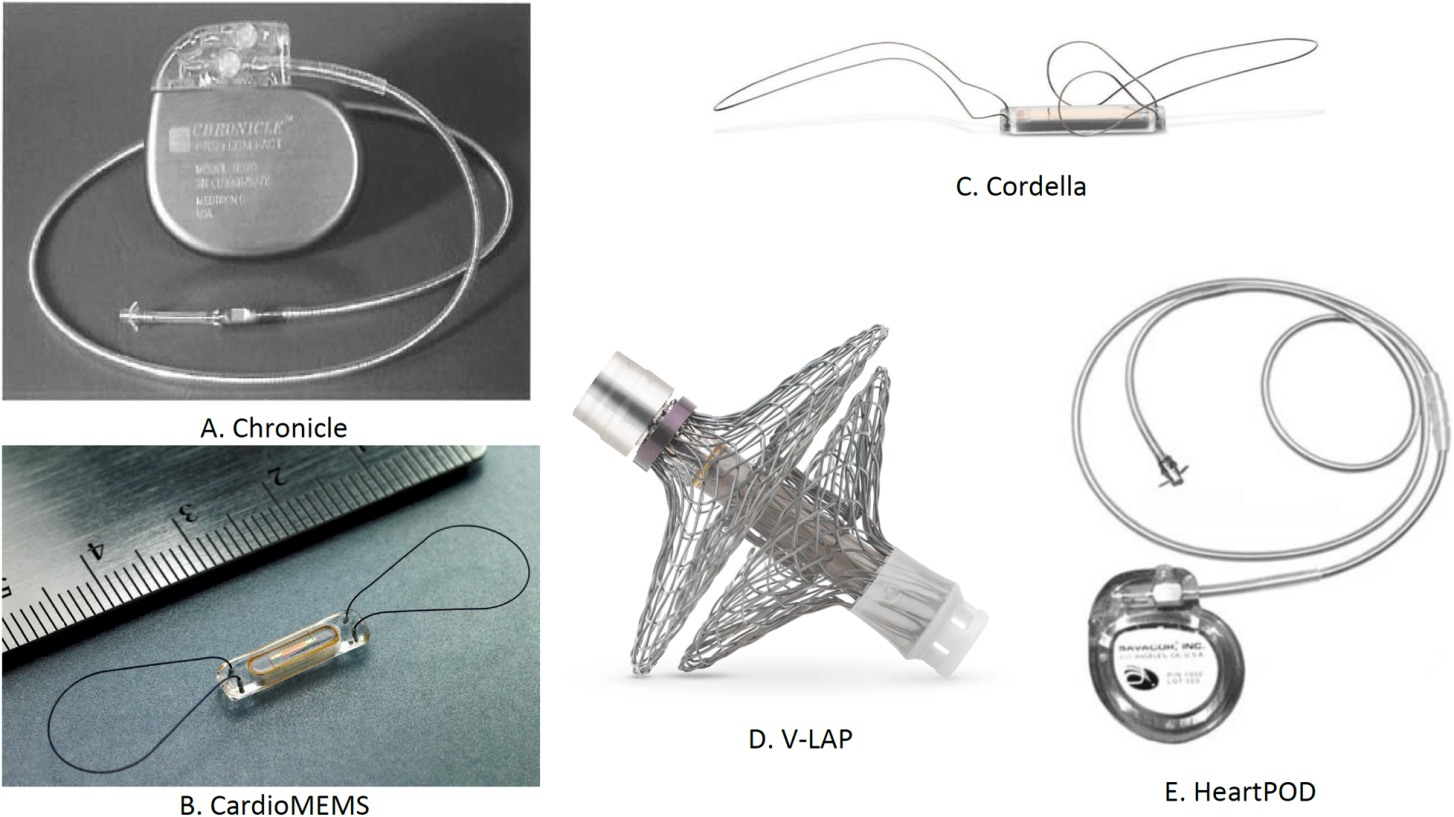
Implantable sensors for hemodynamic monitoring: (**A**) Chronicle, (**B**) CardioMEMS, (**C**) Cordella, (**D**) V-LAP, and (**E**) HeartPOD. ^†^LAP, left atrial pressure.

Remote monitoring of patients with suspected arrhythmia is facilitated by another novel device, i.e., Reveal LINQ (Medtronic, MN, USA), which is a miniaturized insertable cardiac monitor. Reveal LINQ is quite smaller than its predecessor, which majorly utilizes a wireless telemetry system for remote monitoring and is characterized by the presence of a new P-wave filter to refine the atrial fibrillation (AF) algorithm performance (58). A non-randomized, prospective, and multicenter clinical trial, i.e., Reveal LINQ Usability Study enrolling 30 patients, was conducted to demonstrate the efficacy of Reveal LINQ. The findings of the study suggested that, without safety concerns, the miniaturized Reveal LINQ facilitates intensive medical care by detection and monitoring of arrhythmia (59). Various implantable sensors for hemodynamic monitoring are demonstrated in Figure 8.

**Summary**

• **Chronicle**: Implanted in the right ventricle for RA pressure assessment. It is recommended for patients with NYHA Class III or IV HF on standard medical therapy. While no system-related complications were reported, it showed no significant reduction in HF-related adverse events. It was evaluated in the Compass-HF trial (*n* = 247) but is not FDA approved. Clinical trials are ongoing.
• **CardioMEMS**: Placed in a branch of the PA during right heart catheterization to monitor PA pressure. It significantly reduced HF hospitalizations by 58% and all-cause hospitalizations by 28%. Evaluated in the CHAMPION trial (*n* = 500) and GUIDE HF trial (*n* = 1,000), it is FDA approved.
• **Cordella™ PA Pressure Sensor System**: Implanted in the right PA to assess PA pressure. It is recommended for NYHA Class III HF patients with a history of HF hospitalizations. It showed improvements in NYHA functional class and QOL with no device-related complications. Trials include SIRONA (*n* = 15), SIRONA-2 (*n* = 70), and the ongoing PROACTIVE-HF IDE trial. It is not FDA approved, with ongoing trials.
• **V-LAP**: Implanted in the inter-atrial septum to assess LA pressure. It is recommended for patients with NYHA Class III–IVa HF and a history of HF hospitalizations. It showed improvements in NYHA class and 6-MWD with no device-related complications. Ongoing trial: VECTOR-HF.
• **HeartPOD system**: Implanted with a coil antenna and sensor across the atrial septum for LA pressure assessment. It is recommended for patients with NYHA Class II HF and elevated pro-BNP levels. It showed no device-related complications and simultaneous rise in pulmonary capillary wedge pressure. Evaluated in HOMEOSTASIS (*n* = 40) and LAPTOP-HF (*n* = 730), but it is not FDA approved. Clinical trials are ongoing.

The implantation site, mode of action, and clinical findings of the trials conducted with implantable sensors used for hemodynamics monitoring are presented in Table 1.

**Table 1:** Implantable sensors for hemodynamic monitoring.

Name of the device	Implantation site	Mode of action	Major inclusion criteria for patients	Clinical findings	Clinical trials conducted and ongoing	Approval status
Chronicle	Right ventricle	RA pressure assessment	Patients present with NYHA Class III or IV HF, 3 months before enrollment patients must be on standard medical therapy of angiotensin and beta-blockers drugs, 1 incidence of hospitalization due to HF	No system-related complications and pressure failure sensor cases were reported; however, no marked difference was reported in the reduction of HF-related adverse events	Compass-HF trial (*n* = 247)	Not FDA approved Clinical trials ongoing
CardioMEMS	Branch of PA during right RHC	PA pressure investigation	Patients present with NYHA Class VI HF instead of absence or presence of LVEF, within the 12 preceding months, hospitalizations due to HF	Reduction in HF hospitalization per year by 58%, reduction in incidence of all-cause hospitalization by 28%, combined endpoint of HF hospitalization and death decline by 44%	CHAMPION trial (*n* = 500) GUIDE HF trial (*n* = 1000)	FDA approved
Cordella™ PA Pressure Sensor System	Right PA	Assessment of pressure in pulmonary artery	Patients present with NYHA Class III HF, at least one incidence of hospitalization due to HF, presence of preserved EF provided treatment for a 3-month period, in the preceding year need of administration of intravenous (IV) diuretics	NYHA functional class improvement, marked improvement in quality of life of patient as demonstrated by KCCQ, no incidence of device-related complications was reported	SIRONA pilot trial (*n* = 15) SIRONA-2 trial (*n* = 70) PROACTIVE-HF IDE trial (NCT04089059) (Clinical trial ongoing)	Not FDA approved Clinical trials are ongoing
V-LAP	Inter-atrial septum left and right sides	LA pressure assessment	For at least a 6-month period, patient present with NYHA Class III–IVa HF, history of 1 incidence of hospitalization due to HF within the past year, BNU elevated level	Improvements in mean NYHA class and 6-MWD, no device-related complications, device explant, or death was reported	VECTOR-HF (ongoing clinical trial)	Clinical trials are ongoing
HeartPOD system	Coil antenna implanted in subcutaneous pocket and across the atrial septum, sensor pocket is placed	LA pressure assessment	In past 12 months, history of hospitalizations due to HF, NYHA Class II, rise in the level of B-type natriuretic peptide	No device-related complications were seen, Simultaneous rise in Pulmonary capillary wedge pressure	HOMEOSTASIS (*n* = 40) LAPTOP-HF (*n* = 730)	Not FDA approved Clinical trials are ongoing

RA, right atrium; NYHA, New York Heart Association; HF, heart failure; RHC, right heart catheterization; LVEF, left ventricular ejection fraction; IV, intravenous; KCCQ, Kansas City cardiomyopathy questionnaire; LA, left atrium; BNU, brain natriuretic peptide; MWD, minutes walking distance.

The medical field has seen promising advancements in implantable devices for hemodynamic monitoring of patients with HF. However, their continuous evolution and rigorous research are crucial to maximize their efficiency, safety, and positive impact on patient outcomes.

**Present status**

• Remote monitoring with cardiac implantable electronic devices (CIEDs) has not improved HF outcomes due to data limitations, such as low sensitivity of weight and biomarker tracking.
• The Chronicle IHM system reduced HF admissions by 57% through hemodynamic monitoring, particularly focusing on PAP.
• The COMPASS-HF study showed PAP-guided therapy was effective when physicians adjusted treatment based on PAP, although NYHA Class IV patients saw less benefit.
• The CHAMPION trial demonstrated a 37% reduction in HF hospitalizations using daily PAP monitoring, with significant improvements in QOL and low device complications.
• New systems under development include Medtronic's device for PAP, arrhythmias, and heart rate, and Endotronix's system similar to CardioMEMS but with a different interface.
• Devices to monitor LAP are being developed, as LAP directly reflects left ventricular filling pressure and may offer more clinical insights.
• The HeartPOD system showed similar outcomes to CHAMPION in reducing HF hospitalizations, but the LAPTOP-HF trial was stopped early due to implant-related complications.
• The V-LAP system is a next-generation, wireless LAP sensor implanted transseptally, with advanced data analysis capabilities for HF management.
• Another LAP monitoring system by Integrated Sensing Systems requires surgical implantation and is evaluated in patients undergoing cardiac surgery.
• Technologically advanced implantable hemodynamic monitoring systems and novel data use approaches may further improve management of patients with HF in the future (60).

#### 1.3.2 Left atrial decompression devices

During exercise, healthy individuals experience increased stroke volume, CO, and heart rate due to the compliance in ventricles. Contrarily, patients with HF face impaired ventricular compliance, leading to elevated left ventricular end-diastolic pressure (LVEDP). This increase, affecting the LA, results in pulmonary congestion and heightened mortality. Therefore, reducing LVEDP is crucial during treatment.

One notable method is controlled left-to-right intra-arterial shunting using inter-atrial shunt devices (IASDs). These devices help establish inter-atrial communication, counteracting the effects of pulmonary edema (61). Left atrial decompression, which is a novel treatment, reduces mortality by decreasing the LA pressure.

Multiple IASDs, from temporary to permanent solutions, are being evaluated. The examples include the Corvia Inter-Atrial shunt (Corvia, Tewksbury, MA, USA), V-wave system (Johnson and Johnson, Medtech, New Brunswick, NJ, USA), transcatheter atrial shunt system (TASS), and atrial flow regulator (AFR) (Occutech, Schaffhausen, Switzerland). The Corvia device, which is designed for HFpEF, showed initial positive results (62). V-wave's device displayed promising preliminary results in reducing the LA pressure (63). The various shunt sizes of AFR resulted in clinical improvements, and TASS led to reduced HF-related hospitalizations and enhanced QOL.

**Figure 9:**
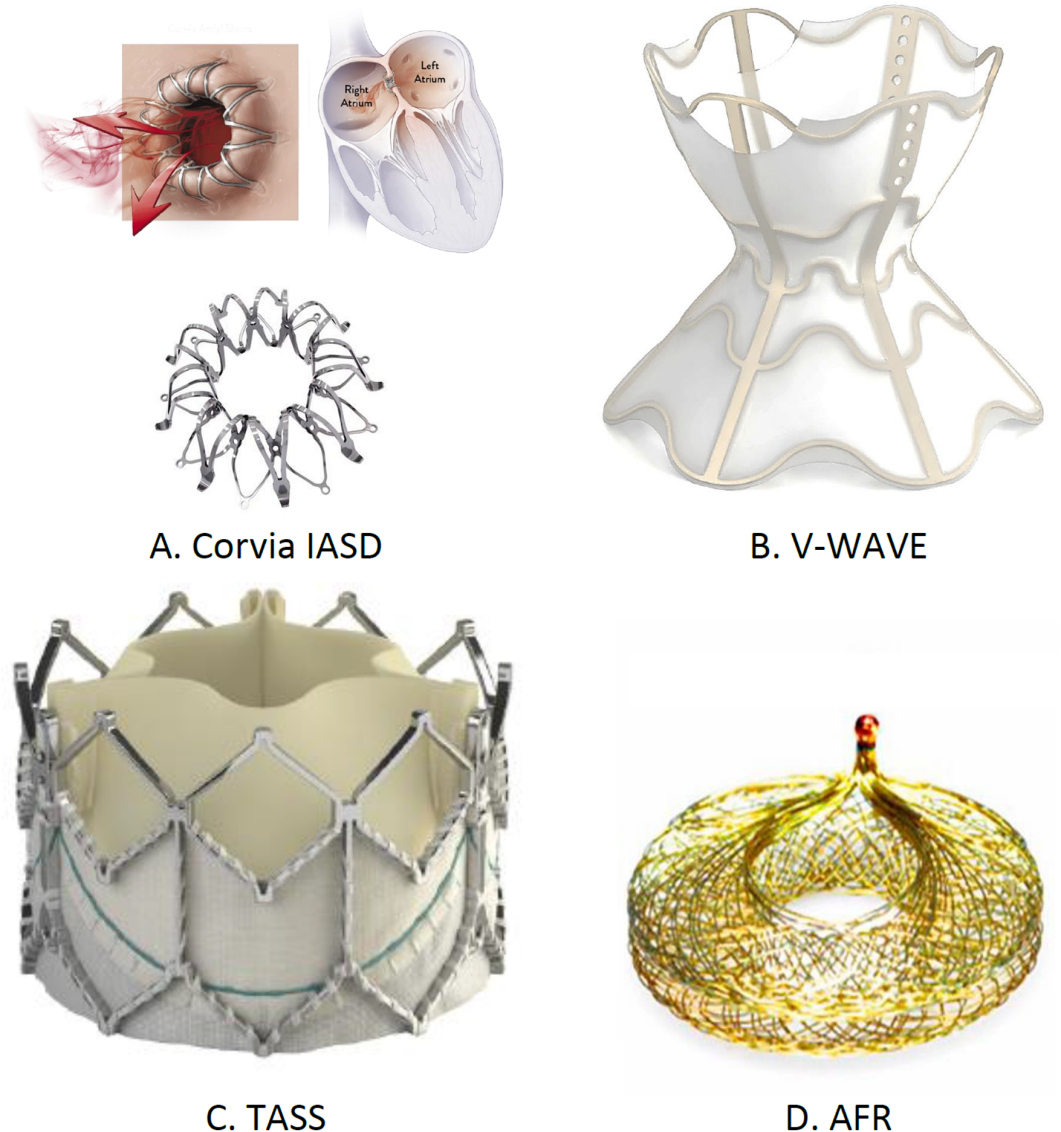
Implantable left atrial decompression devices: **(A)** Corvia IASD, **(B)** V-wave, **(C)** TASS, and **(D)** AFR. ^†^IASD, inter-atrial shunt device; TASS, transcatheter atrial shunt system; AFR, atrial flow regulator.

The NoYa system, a stentless shunt using radiofrequency energy, avoids permanent implants, with early data indicating patient benefits (64). Alleviant's unique design ensures shunt patency without utilizing permanent stents and has shown encouraging initial results (65). To establish the lasting effectiveness and safety of these devices, conducting comprehensive trials is essential. Various left atrial decompressive devices to decompress the LA pressure are shown in Figure 9.

**Summary**

• **CORVIA IASD**: Implanted in the intra-atrial septum to create a shunt from left to right. Targets patients with symptomatic HF, EF <40%, high pulmonary capillary wedge pressure (PCWP) during exercise, and NYHA Class II–IVa. Clinical findings show improvements in NYHA class but no significant reduction in HF hospitalizations or cardiovascular death. Trials: REDUCE-LP-HF and reduce elevated left atrial pressure in patients present with heart failure I. Not FDA approved; trials ongoing.
• **V-wave**: Implanted at the inter-atrial septum to reduce LA filling pressure by shunting blood from the left atrium to the right. Targets patients with NYHA Class III–IV HF and a history of HF hospitalizations. Shows improvements in NYHA class and high Kansan City Cardiomyopathy Questionnaire (KCCQ) scores. Trials: VW-SP-1, Canadian cohort, and RELIEVE-HF. FDA approved.
• **TASS**: Implanted via the right internal jugular vein, shunting blood from left atrium to right atrium. It is recommended for patients on GDMT with NYHA Class II–IVa HF, and elevated PCWP. Shows improvements in KCCQ scores, 6-MWD, and NYHA class. Trials: TCT-87 and NCT03523416. Not FDA approved; trials ongoing.
• **Atrial flow regulator**: Implanted in the intra-atrial septum to create a shunt from left to right. It is recommended for patients with HFpEF, NYHA Class II–IVa, and elevated PCWP. Shows improvements in NYHA class, 6-MWD, and KCCQ scores. Trial: NCT05136820. Not FDA approved; trials ongoing.

The mode of action, implantation site, and clinical findings of trials using left atrial decompressive devices are presented in Table 2.

**Table 2:** Left atrial decompression devices.

Name of the device	Implantation site	Mode of action	Major inclusion criteria for patients	Clinical findings	Clinical trial conducted	FDA approval
CORVIA IASD	Left to right intra-arterial shunt	Intra-arterial shunting from left to right, implantation in permanent manner	Patient present with symptomatic HF, less than 40% EF, during exercise PCWP greater than 25 mmHg, more than 5 mmHg right arterial pressure, NYHA Class II–IVa, rise in the level of BNP, in the preceding year at least one incidence of hospitalization due to HF	Marked improvements in NYHA functional class. No difference in hospitalizations due to HF or cardiovascular death was reported. High KCCQ score was reported	REDUCE-LP-HF NCT01913613 (*n* = 64) REDUCE-LAP-HF I NCT02600234 (*n* = 44) ongoing clinical study	Not FDA approved Clinical trials are ongoing
V-wave	At the level of inter-atrial septum	Reduce LA filling pressure by blood volume shunting from left atrium to right	In the preceding year, at least one incidence of HF hospitalization, rise in the level of BNP, presence of HF in patients with NYHA Class III–IV ventriculoarterial (VA).	Improvement in NYHA class from III to II. High KCCQ scores	VW-SP-1+ Canadian cohort8 (*n* = 38) RELIEVE-HF NCT03499236 (*n* = 500) ongoing clinical study	FDA approved
TASS	Right internal jugular vein	From left atrium to right atrium, intra-arterial shunting	For at least 3 months use of GDMT, in the preceding year at least one incidence of hospitalization due to HF, patients present with HF with NYHA Class II–IVa, more than 15 mmHg PCWP during rest or during exercise PCWP more than 25 mmHg	KCCQ scores and 6-MWD score improvements, NYHA functional class improvements	TCT-87 (*n* = 11 patients NCT03523416 (n-75) (early feasibility study ongoing)	Not FDA approved clinical trials are ongoing
Atrial flow regulator	Intra-arterial septum	Intra-arterial shunting from left to right	Patients present with symptoms HFpEF, NYHA Class II–IVa HF with EF ≥15% rise in the PCWP. At rest PCWP reported <15 mm/hg and more than 25 mmHg during exercise, in the preceding year incidence of HF hospitalization	Marked improvements in NYHA class, improvement in 6-MWD and KCCQ score	NCT05136820 (*n* = 698)	Not FDA approved clinical trials are ongoing

PCWP, pulmonary capillary wedge pressure; IASD, inter-atrial shunt devices; BNP, brain natriuretic peptide; NYHA, New York Heart Association; HF, heart failure; KCCQ, Kansas City cardiomyopathy questionnaire; LA, left atrium; TASS, transcatheter atrial shunt system; 6-MWD, 6-minute walking distance.

**Present status:**

• LA function is increasingly important in HF management, especially for HFpEF, where reducing LA size and pressure are crucial.
• Invasive procedures to measure LAP carry risks, including thrombosis, requiring careful patient selection and anticoagulation.
• Current LA devices under development have not received FDA approval, and their impact on arrhythmia prevention or treatment is unclear.
• Remote LAP monitoring showed potential during the corona virus disease 2019 pandemic for preventing HF decompensation, indicating a growing role for such technologies.
• The Corvia Atrial Shunt Device, despite the FDA breakthrough designation, failed in long-term efficacy trials, while pump-based devices are still in early experimental stages (66, 67).

#### 1.3.3 LV restoration devices

**Figure 10:**
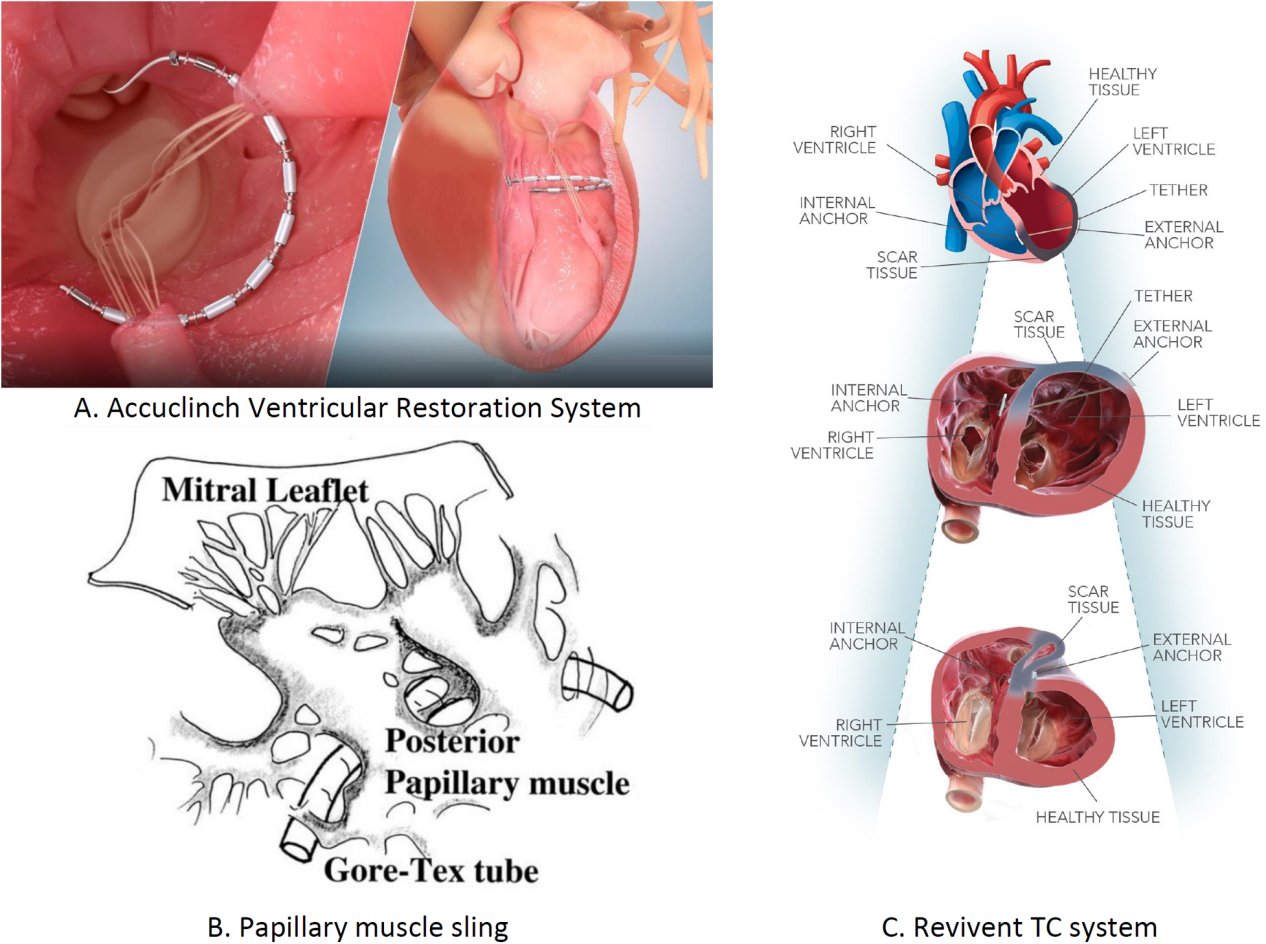
Left ventricular restoration devices: **(A)** AccuClinch ventricular restoration system, **(B)** papillary muscle sling, and **(C)** Revivent TC system.

LV dysfunction arises from the molecular and cellular changes due to pathological stress. Ventriculoplasty addresses this by reducing the LV volume and enhancing patient outcomes. The notable methods include the papillary muscle sling, Revivent TC system, and AccuClinch. Originally used for functional mitral regurgitation (FMR), AccuClinch is now utilized to treat HFrEF, positioning the LV's basal aspects (68). Its effectiveness will be evaluated by the ongoing CORCINCH-HF trial. The papillary muscle sling technique, which reduces inter-papillary muscle distance, is under trial. The Bioventrix Revivent TC system, a hybrid transcatheter, removes the LV scars. In selected patients with ischemic cardiomyopathy, the Bioventrix Revivent TC system facilitates LV reshaping and scar exclusion. This system is majorly designed to potentiate reverse remodeling and improve QOL (69). The various LV restoration devices used for performing ventriculoplasty are in Figure 10.

**Summary**

• **AccuClinch ventricular restoration system (Ancora, Cleveland, OH, USA)**: Implanted in the LV sub-valvular space to reduce the diameter of the basal wall and LV volume. Targets patients with left ventricular end-diastolic diameter (LVEDD) >55 mm, LVEF between 20% and 40%, and NYHA Class II–IV. No clinical evidence yet as trials are ongoing. Trial: CORCINCH-HF (*n* = 400). Not FDA approved; trials ongoing.
• **Papillary muscle sling**: Implanted at the base of the small posterior papillary muscle to reduce the lateral distance between inter-papillary muscles. It is recommended for patients with ischemic or non-ischemic cardiomyopathy, inter-papillary muscle distance >20 mm, and NYHA Class II–IV. No clinical evidence yet as trials are ongoing. Trial: NCT04475315 (*n* = 40). Not FDA approved.
• **Revivent TC system (Bioventrix, San Raman, CA, USA)**: Implanted in the LV for reconstructing an abnormally dilated LV by excluding scars in the LV apical wall. Recommended for patients with HF symptoms, history of MI, and elevated LV systolic volume. At 6-month follow-up, improvements in QOL, 6-MWT, LVEF, and NYHA class were noted. Trials: REVIVE-HF (*n* = 180), NCT0384512. Not FDA approved; trials ongoing.

The mode of action, implantation site, and clinical findings of trials conducted on LV restoration devices are shown in Table 3.

**Table 3:** Left ventricular restoration devices.

Name of the device	Implantation site	Mode of action	Major inclusion criteria for patients	Clinical findings	Clinical trials conducted/ongoing	FDA approval
AccuClinch ventricular restoration system	LV sub-valvular space	Reducing the diameter of basal wall and volume of LV	More than 55 mm LVEDD, in between 20 and 40% LVEF, 6-minute walk distance 100–450 m, NYHA Class II–IV	No clinical evidence as clinical trial is ongoing	CORCINCH-HF (*n* = 400) ongoing clinical study	Not FDA approved Clinical trials are ongoing
Papillary muscle sling	Small posterior papillary muscle base	Reducing the lateral distance in between inter-papillary muscles	Patients present with ischemic or non-ischemic cardiomyopathy, more than 20 mm inter-papillary muscle distance, in between 20% and 50% LVEF, LVEDD more than 55, NYHA Class 11–IV	No clinical evidence as clinical trial is ongoing	NCT04475315 (*n* = 40) ongoing clinical study	Not FDA approved Clinical studies are ongoing
Revivent TC system	Left ventricular	Reconstruction of abnormally dilated LV by excluding scar present in LV apical wall	Symptoms of heart failure with previous history of MI, rise in the LV systolic volume	At the follow-up of 6 months, marked change in various parameters like QOL, 6-MWD, LVEF, and NYHA class occurred	REVIVE-HF (*n* = 180) NCT0384512	Not FDA approved clinical trials are ongoing

LV, left ventricular; LVEDD, left ventricular end-diastolic diameter; LVEF, left ventricular ejection fraction; NYHA, New York Ejection Fraction; MI, myocardial infarction; QOL, quality of life; 6-MWD, 6-minute walking distance.

Despite the advancements in MI treatments, post-injury LV remodeling often progresses to HF. The key indicators of adverse LV remodeling, including LVEF and LV dimension, are correlated with increased post-MI cardiovascular incidence and mortality rates. Interventions, whether drug or device-based, targeting LV remodeling have critically reduced mortality from MI (70).

Several surgical and transcatheter methods aim to modify the LV shape post-MI. Notably, the parachute device effectively limits LV dimensions in patients with ischemic HF, as shown in the PRACHUTE trial (71). In managing FMR, Coapsys™ reduces muscle displacement, especially among patients who had undergone coronary artery bypass graft (CABG), whereas its variant, iCoapsys™, maintains the design integrity with a unique implant approach. A randomized controlled study, RESTOR-MV, involving patients referred for CABG with FMR demonstrated its efficacy. This study involving 165 patients, with the iCoapsys™ device showing a marked survival rate compared with control at 2 years (87% vs. 77%) (hazard ratio: 0.421; 95% confidence interval: 0.200–0.886; stratified log-rank test; *p* = 0.038) (72).

Numerous transcatheter solutions address the mitral apparatus. The MitraClip demonstrated reduced LA volume in patients with ischemic FMR, but it left the LV volume unchanged (73). The Monarc system was also effective, but some patients developed complications (74). The percutaneous transvenous mitral annuloplasty device, designed for chronic FMR, showed promising results in the PTOLEMY-2 study (75). The Carillon Mitral Contour System led to considerable reductions in the regurgitant volume and improved 6-MWD test outcomes across trials (76). Lastly, the innovative mitral loop cerclage reduced the regurgitant volume and LVEDP in the early tests.

#### 1.3.4 Neuromodulation for the management of HF

**Figure 11:**
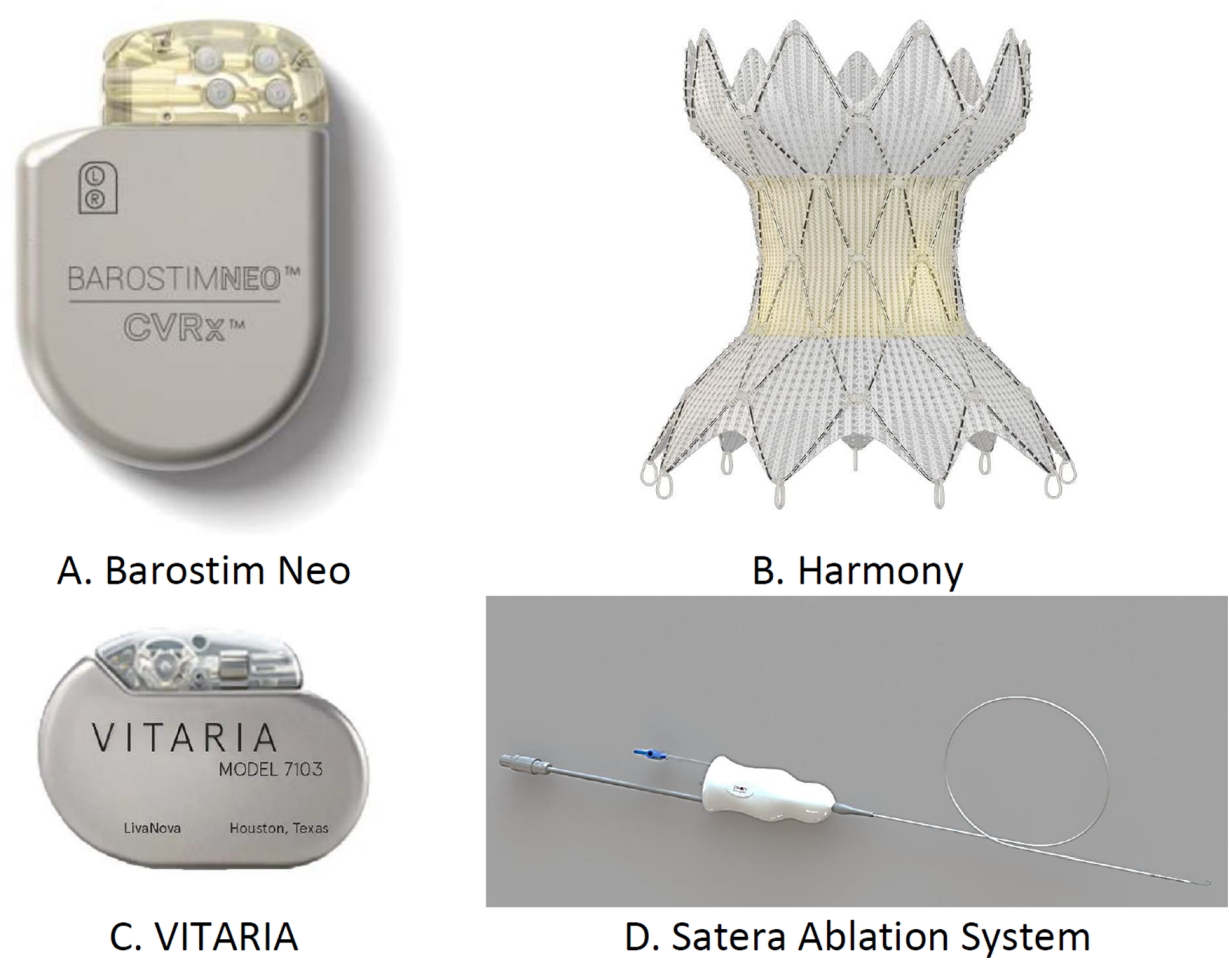
Transcatheter devices used for neuromodulation: **(A)** Barostim Neo, **(B)** Harmony system, **(C)** Vitara, and **(D)** Satera ablation system.

In patients with HF, autonomic imbalance from increased sympathetic tone and reduced parasympathetic activity heightens the myocardial workload, worsening the HF symptoms. The devices target this imbalance, focusing on the parasympathetic system or baroreceptors (77). Barostim NEO (CVRx) stimulates the carotid baroreceptors, reducing the sympathetic activity. The HOPE4HF and BeAT-HF (78) studies confirmed its benefits, showing improvements in LVEF, 6-MWD, and NYHA classification. The Harmony system, targeting the aortic wall, is under the ENDO-HF study (79). The VITARIA system, affecting the vagus nerve, improved the LVEF, 6-MWD outcomes, and NYHA functional class in the autonomic neural regulation therapy to enhance myocardial function in heart failure study (80). Splanchnic nerve blockade (SNB) induces vasodilation, reducing pre-load and afterload (81). The Satera Ablation System, designed for SNB, demonstrated cardiac enhancements in both surgical (82) and percutaneous applications (83), with a major trial (NCT04592445) currently in progress. The various transcatheter devices used for neuromodulation in patients present with HF are shown in Figure 11.

**Summary**

• **Barostim Neo**: Electrodes are implanted in the carotid artery area to reduce sympathetic activity and increase parasympathetic activity by activating baroreceptors. It is recommended for patients over 21 years old with EF <35%, NYHA Class II–III, and on GDMT for at least 1 month. Clinical trials (BeAT-HF, *n* = 408; Barostim HOPE4HF, *n* = 98) show improvements in BNP levels, QOL, and 6-MWD. FDA approved.
• **Harmony system (Medtronic, Minneapolis, MN, USA)**: Implanted endovascularly into the thoracic aorta to increase parasympathetic activity and reduce sympathetic activity. It is recommended for patients with NYHA Class II–III on optimal GDMT. Clinical findings are pending; ongoing trial (ENDO-HF, *n* = 30). FDA approved.
• **Vagal nerve stimulation (VITARIA, Electrocore, Basking Ridge, NJ, USA)**: Pectoral pulse generator with electrode placed over the vagus nerve to reduce sympathetic activity and increase parasympathetic activity. It is recommended for patients with LVEF <40%, 5–8 cm LVEDD, NYHA Class II–III. Trials (ANTHEM-HFrEF, *n* = 533) show improvements in LVEF, NYHA class, QOL, and 6-MWD. Not FDA approved.
• **SATERA ablation system (Axon Therapies, New York, NY, USA)**: Catheter ablation of the splanchnic nerve at the 10th–11th thoracic vertebrae to redistribute intra-vascular volume. Targets patients on GDMT for more than 1 month, with LVEF <50%, NYHA Class II–IVa. Clinical findings are pending; ongoing trial (NCT04592445). Not FDA approved.

The implantation site, mode of action, and clinical trials conducted with the devices used for neuromodulation in patients with HF are presented in Table 4.

**Table 4:** Devices used for neuromodulation for HF.

Name of the device	Implantation site	Mode of action	Major inclusion criteria for patients	Clinical findings	Clinical trials conducted/ongoing	Approval status
Barostim Neo	Electrodes are placed in carotid artery area	Reduction in sympathetic activity and increase in parasympathetic activity by activating the baroreceptors	Patient age more than 21 years, at least for 1 month patient has been on GDMT, less than 35% EF, NYHA Class III–11	Improvements in various parameters like rise in the level of pro-BNP, QOL, and 6-MWD	BeAT-HF (*n* = 408) Barostim HOPE4HF (*n* = 98)	FDA approved
Harmony system	Into the thoracic aorta, endovascular implantation	Rise in parasympathetic activity and reduction in sympathetic activity by activating the	On optimal GDMT patient present with NYHA Class II–III	Clinical study is going on. Clinical findings are pending	ENDO-HF (*n* = 30) Ongoing clinical study	FDA approved
Vagal nerve stimulation (VITARIA)	Over the vagus nerve, placement of pectoral pulse generator with electrode	Reduction in sympathetic activity and rise in parasympathetic activity	Less than 40% LVEF, 5–8 cm LVEDD and NYHA Class II–III	Marked improvements in various parameters LVEF, NYHA functional class, QOL, and 6-MWD	ANTHEM-HFrEF (*n* = 533)	Not FDA approved
SATERA Ablation system	In the 10th–11th thoracic vertebrae, splanchnic nerve catheter ablation	Redistribution of intra-vascular volume by blocking the splanchnic nerve	At least for more than 1 month patient has been on GDMT, less than 50% LVEF, NYHA Class 11–IVa, more than 25	Clinical findings are pending as clinical trial study is going on	NCT04592445 (clinical trial ongoing on)	Not FDA approved Clinical trial study are ongoing

GDMT, guidelines-directed medical therapy; BNP, brain natriuretic peptide; QOL, quality of life; 6-MWD, 6-minute walking distance; NYHA, New York Heart Association; LVEDD, left ventricular end-diastolic diameter; LVEF, left ventricular ejection fraction.

**Present status**

• Current guidelines from the ACC, AHA, and ESC do not provide strong recommendations for neuromodulation in chronic HF due to limited evidence.
• Neuromodulation devices, including BAT and renal denervation (RDN), recognized for potentially improving symptoms and QOL, lack robust data on mortality benefits.
• The Barostim NEO is the only FDA-approved neuromodulation device for advanced HF, showing significant improvements in QOL, 6MWD, and NT-pro-BNP levels in the BeAT-HF trial.
• The ESC mentions the modest benefits of BAT but notes the need for more data on hard clinical outcomes like mortality.
• RDN is highlighted for its potential in treating hypertension and left ventricular hypertrophy, although further research is required (84–86).

#### 1.3.5 Device-based therapy for cardiorenal syndrome

**Figure 12:**
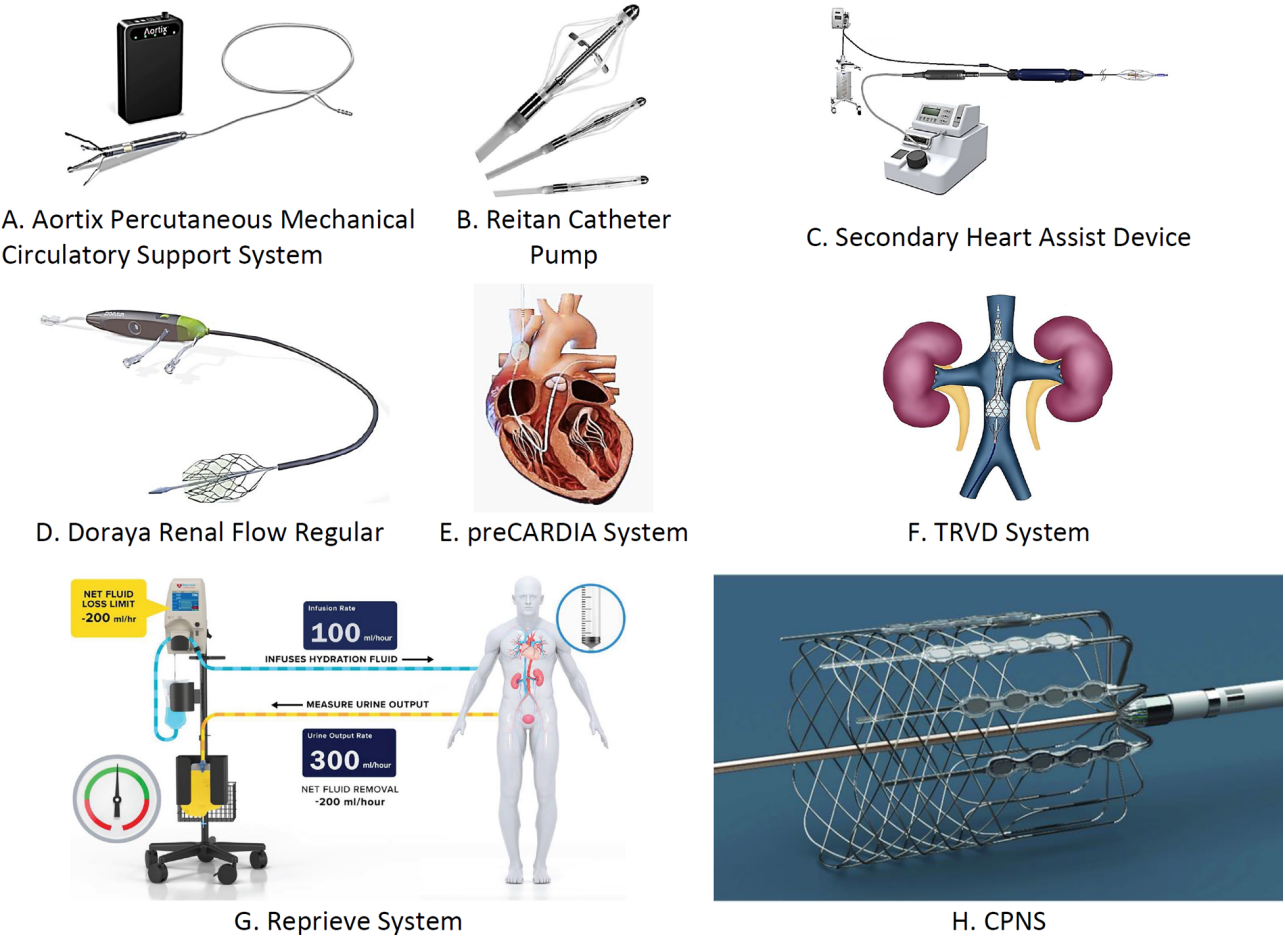
Transcatheter devices used for the management of acute cardiorenal syndrome: **(A)** Aortix percutaneous mechanical circulatory support system, **(B)** Reitan catheter pump, **(C)** secondary heart assist device, **(D)** Doraya renal flow regulator, **(E)** preCARDIA system, **(F)** TRVD system, **(G)** reprieve system, and **(H)** CPNS. ^†^TRVD, transcatheter renal venous decongestion; CPNS, cardiac pulmonary nerve stimulation.

Patients with acute decompensated HF (ADHF) often exhibit reduced CO, leading to hypotension and RAAS pathway activation. This further results in vasoconstriction, lowered renal perfusion, and increased diuretic resistance (83), making ADHF challenging to treat. Renal venous congestion exacerbates these conditions. To address this, catheter-based devices have been developed to alleviate venous congestion and boost renal perfusion. Figure 12 illustrates the devices for managing cardiorenal syndrome (CRS), and Table 5 provides detailed information on these devices.

**Table 5:** Various devices used for management of cardiorenal syndrome.

Name of the device	Implantation site	Mode of action	Major inclusion criteria for patients	Clinical findings	Clinical trials conducted	FDA approval
Aortix percutaneous mechanical circulatory support system	Implanted through the femoral artery and positioned in the descending aorta	Reduce cardiac afterload and potentiate renal perfusion	More than 20 PCWP, central venous pressure less than 12, hospitalizations with renal dysfunctioning and ADHF	No incidence of device-related complications was reported, modulates the significant rise in the urine output	Aortix CRS pilot study (*n* = 30)	NOT FDA approved Clinical studies are ongoing
Reitan catheter pump	Implanted through the femoral artery and positioned in the proximal descending aorta	Potentiate renal perfusion and decrease the cardiac afterload	N/A	Findings of efficacy studies demonstrate about the improvement in urine output, cardiac index, and serum creatinine with this pump	TCT-CONNECT 179 (*in vivo* safety studies)	Not FDA approved Clinical studies are ongoing
Second Heart Assist Device	Implanted through the femoral artery and positioned in the proximal descending aorta	Reduce the cardiac afterload and modulate the renal perfusion	N/A	Till date no clinical evidence was reported with this device	No clinical trial still conducted	Not FDA approved Clinical studies are ongoing
Doraya renal flow regulator	Inserted through the femoral vein	Potentiate the renal perfusion and reduce the cardiac afterload	Patients present with ADHF and do not respond to diuretics, 15%–35% LVEF, CVP must be less than 12 mmHg, GFR (glomerular filtration must be between 20 and 40)	Significant increase in urine output, no incidence of serious complications associated with the device insertion was reported	NCT03234647 (*n* = 9)	Not FDA approved Clinical studies are ongoing
PreCARDIA system	Inserted into superior vena cava at the junction of right atrium	Lead to reduction in cardiac afterload and modulate the renal perfusion rate	Patients present with NYHA Class III–IV who do not respond to diuretics	Concept study findings with huge decline in bioventricular pressures	VENUS-HF (*n* = 30) (early feasibility study)	FDA approval
TRVD system	Inserted into the femoral vein	Reduction in cardiac afterload and increase the renal perfusion rate, distension of IVC	Patients present with ADHF, more than 14 mmHg CVP, rise in the level of BNP, less than 40% LVEF	Decline in CVP, reduction in level of BNP, reduction in creatinine level	NCT03621436 (*n* = 130)	Not FDA approved Clinical studies are ongoing
Reprieve system	Through a peripheral vein, fluid management device is connected to patient	Evaluating patient's urine output, pre-define sterile replacement solution administration to maintain fluid balance	Patient diagnosed with ADHF, patient present with history of chronic renal failure, patient present with certain symptoms like persistent dyspnea at rest, pulmonary congestion	Marked rise in diuresis, reduction in central venous pressure, drop in mean creatinine level, no change in renal injury biomarkers	TARGET-1 (*n* = 10) and TARGET-2 studies (*n* = 9) (NCT03897842)	FDA approved
CPNS	Catheters were implanted in LV and aortic root	Stimulation of cardiopulmonary plexuses	Implantation of cardiac resynchronization therapy for HF management, patient is on oral pharmacological standard treatment for HF management, patient present with LVEF <35%	Marked rise in systolic pressure, diastolic pressure, mean atrial pressure, and left ventricular systolic pressure were seen	First-in-human study (*n* = 15)	Not FDA approved Clinical trials are ongoing

GDMT, guideline-directed medical therapy; ADHF, acute decompensated heart failure; LVEF, left ventricular ejection fraction; CVP, central venous pressure; GFR, glomerular filtration rate; NYHA, New York Heart Association; TRVD, transcatheter renal venous decongestion; CPNS, cardiac pulmonary nerve stimulation; IVC, inferior vena cava.

**Summary**

• **Aortix**: Implanted via femoral artery in the descending aorta to reduce cardiac afterload and boost renal perfusion. It is recommended for patients with PCWP >20, CVP <12, ADHF with renal dysfunction. No complications; increased urine output. Aortix CRS study (*n* = 30). Not FDA approved; trials are ongoing.
• **Reitan pump**: Positioned in the proximal descending aorta to enhance renal perfusion and reduce cardiac afterload. Shows improved urine output, cardiac index, and serum creatinine. TCT-CONNECT 179 study. Not FDA approved; trials are ongoing.
• **Second heart assist (Second Hearts Whisper, Salt Lake city, UT, USA)**: Implanted via femoral artery to reduce cardiac afterload. No clinical evidence yet. No trials conducted. Not FDA approved.
• **Doraya**: Inserted through the femoral vein to enhance renal perfusion and reduce cardiac afterload. Recommended for patients with ADHF unresponsive to diuretics. Increased urine output, no serious complications. NCT03234647 (*n* = 9). Not FDA approved; trials are ongoing.
• **PreCARDIA (Abiomed, Danvers, MA, USA)**: Inserted into superior vena cava to reduce cardiac afterload. Recommended for NYHA Class III–IV patients. Significant pressure reduction in early study (VENUS-HF, *n* = 30). FDA approved.
• **TRVD**: Implanted via femoral vein to reduce cardiac afterload and distend IVC. It is recommended for patients with ADHF with high CVP and BNP. Reduced CVP, BNP, creatinine. NCT03621436 (*n* = 130). Not FDA approved; trials are ongoing.
• **Reprieve**: Fluid management via peripheral vein for ADHF with renal failure. Increased diuresis, reduced CVP, creatinine. TARGET-1, TARGET-2 studies. FDA approved.
• **CPNS**: Catheters in LV/aortic root to stimulate cardiopulmonary plexuses for patients with HF on CRT. Early study (*n* = 15) shows pressure rise. Not FDA approved; ongoing trials.

##### 1.3.5.1 Devices for renal artery perfusion (pushers)

The Aortix™ system (Procryion, Houston, TX, USA) enhances renal perfusion and temporarily unloads the cardiac chambers. In a study involving six patients with HFrEF, no major bleeding or hemolysis was noted, but device-related issues arose. The Reitan catheter pump (Cardiobridge, Lotzenacker, Germany), designed for CRS management, was tested in 20 patients with EF of <30%. Post implantation, no major complications were observed (87). The second heart assist device (Second Heart's Whisper, Salt Lake City, UT, USA) is implanted percutaneously into the descending aorta above the renal arteries to eliminate the stroke risk, which potentiates CO and reduces cardiac filling pressures. It is quite an efficient pump as it is able to provide clinical benefit to both the kidneys and heart (88). However, this device, with two designs for severe HF, lacks support based on clinical evidence.

##### 1.3.5.2 Devices for renal afterload reduction (pullers)

The Doraya Renal Flow Regulator (Revamp Medical, Netanya, HaMerkaz, Israel), designed for patients with diuretic-resistant ADHF, reduces renal afterload and cardiac pre-load (89). Approved by the FDA in 2020, it is currently being studied. The preCARDIA system (Abiomed, Danvers, MA, USA), which is aimed to decrease venous congestion, has shown potential in reducing renal afterload and cardiac pre-load. A previous study revealed reduced bioventricular pressure without adverse events in patients with HFrEF (90). The transcatheter renal venous decongestion system, with a propeller-type pump, showed reduced venous pressure and enhanced kidney blood flow in its initial human trial (91).

**Present status**

• New circulatory renal assist devices are being developed to treat acute (type I) CRS.
• “Pushers” are devices designed to enhance renal arterial perfusion, improving renal pre-load.
• “Pullers” aim to alleviate renal venous congestion, reducing renal afterload.
• The effectiveness of CRS devices will depend on their safety and consistent, significant improvements in renal function.
• These devices should also demonstrate durable benefits in clinical outcomes and hemodynamic congestion markers (92).

##### 1.3.5.3 Device-based therapies for enhancing salt volume homeostasis

###### 1.3.5.3.1. Reprieve system (Milford, CT, USA)

Owing to salt restrictions, chloride sensing by the distal nephron macula densa decreases, creating a sodium-avid kidney state. The Reprieve system, developed as a device-based solution, measures the urine output and delivers predefined sterile solution volumes. It minimizes the net fluid loss and compensates for excess fluid loss, reducing the risk of adverse compensatory mechanisms of the kidney, such as sodium and water retention. An early study has indicated its safety and effectiveness in managing diuresis and improving patient outcomes (93).

**Summary**

• Acute heart failure (AHF) congestion is primarily managed with loop diuretics, although optimal dosing regimens are not well established.
• Overdiuresis can lead to harmful effects, including hemodynamic issues and renal injury, leading to cautious diuretic use.
• The Reprieve System is designed to maintain fluid balance during diuretic therapy, preventing excessive fluid loss and protecting renal function.
• The system continuously monitors urine output and adjusts hydration fluid infusion to maintain a set fluid balance.
• The TARGET-1 and TARGET-2 trials are investigating the safety and potential efficacy of the Reprieve System in patients with AHF (93).

###### 1.3.5.3.2 Device-based neuromodulation in acute CRS

Cardionomics, Inc., developed the Cardiac Pulmonary Nerve Stimulation (CPNS) system (Cardionomics, New brighton, MN, USA), a single-use neuromodulation catheter. When used in patients with HF with ADHF, it stimulates the cardiopulmonary plexus in the right pulmonary artery. An early study revealed improved LV contractility, relaxation, and blood pressure in 15 patients with reduced EF who were using the CPNS system (94).

###### 1.3.5.3.3 Other therapeutic interventions for CRS management

Beyond the CPNS, the other interventions for CRS are being explored. CPNS can enhance renal perfusion and regulate myocardial contractility. The emerging WhiteSwell system is designed to boost lymphatic flow. It is composed of a catheter with an impeller pump that potentiates lymphatic drainage. Further, an area of low pressure is created by the catheter, where the thoracic duct connects with the venous system present near the heart. A proof-of-concept study to demonstrate the efficacy of the WhiteSwell system was conducted by Abraham et al. using a sheep model of HF (95). The management of HF is summarized in the Central illustration.

## 2 Conclusion

Device therapy for HF has seen marked advancements, offering new treatment avenues for those unresponsive to guideline-directed medical therapy. The success of these interventions hinges on precisely identifying the pathophysiological targets and tailoring treatments against guideline-directed medical protocols. Moreover, the trial standards for these devices should align with those used in transcatheter valvular interventions, ensuring that the trial designs understand the device's mechanism and target the HF phenotype. Creating clinical devices poses more challenges than developing drugs. These devices must not only be effective but also safe for long-term use, with a clear net clinical benefit. In summary, there is currently a rising trajectory in the evolution of transcatheter devices. This could supplement or replace medical treatments, addressing the intricate mechanisms of disease pathogenesis. They hold promise in managing various stages of HF, from acute cases to preventing the development of acute decompensation in chronic HF or modifying chronic HF progression.
